# Assessing the consequences of recent climate change on World Heritage sites in South Greenland

**DOI:** 10.1038/s41598-024-60397-9

**Published:** 2024-04-28

**Authors:** Jørgen Hollesen, Malte Skov Jepsen, Martin Stendel, Hans Harmsen

**Affiliations:** 1 Environmental Archaeology and Materials Science, The National Museum of Denmark, IC Modewegsvej, Brede, 2800 Lyngby, Denmark; 2 Danish Meteorological Institute (DMI), Sankt Kjelds Plads 11, 2100 Copenhagen Ø, Denmark; 3 Greenland National Museum & Archives, Hans Egedesvej 8, Boks 145, 3900 Nuuk, Greenland

**Keywords:** Climate-change impacts, Environmental impact

## Abstract

In the Arctic region, microbial degradation poses a significant threat to the preservation of archaeological deposits, actively consuming irreplaceable cultural and environmental records. In this study we assess the potential effects of the last 40 years of climate change on organic archaeological deposits within the UNESCO World Heritage area Kujataa in South Greenland. We use the dynamic process-oriented model, CoupModel to simulate soil temperatures and soil moisture contents at four archaeological sites in the area. The results show that the organic deposits have experienced a substantial warming the last 40 years, which combined with decreasing soil moisture contents creates a dangerous combination that can accelerate the degradation of organic materials. Currently, there are 583 archaeological sites registered within the area. Our findings highlight that the current climatic conditions are not conducive to organic preservation. The greatest risk of degradation lies within the relatively dry continental inland areas of the study region, where all Norse Viking Age settlements are situated. However, even at the "cold" and "wet" outer coast, the combined effects of rising summer temperatures and declining soil moisture levels may already be exerting a noticeable impact.

## Introduction

Climate change is influencing archaeological sites and landscapes on a global scale^1,2^, with a particularly significant impact in the Arctic^3,4^. A growing number of the region's ancient sites and structures are currently deteriorating. Once destroyed, these resources are irreversibly lost. One of the most noteworthy consequences of climate change is the increased microbial degradation of organic archaeological deposits due to rising soil temperatures and shifting hydrology^5–9^.

**Figure 1:**
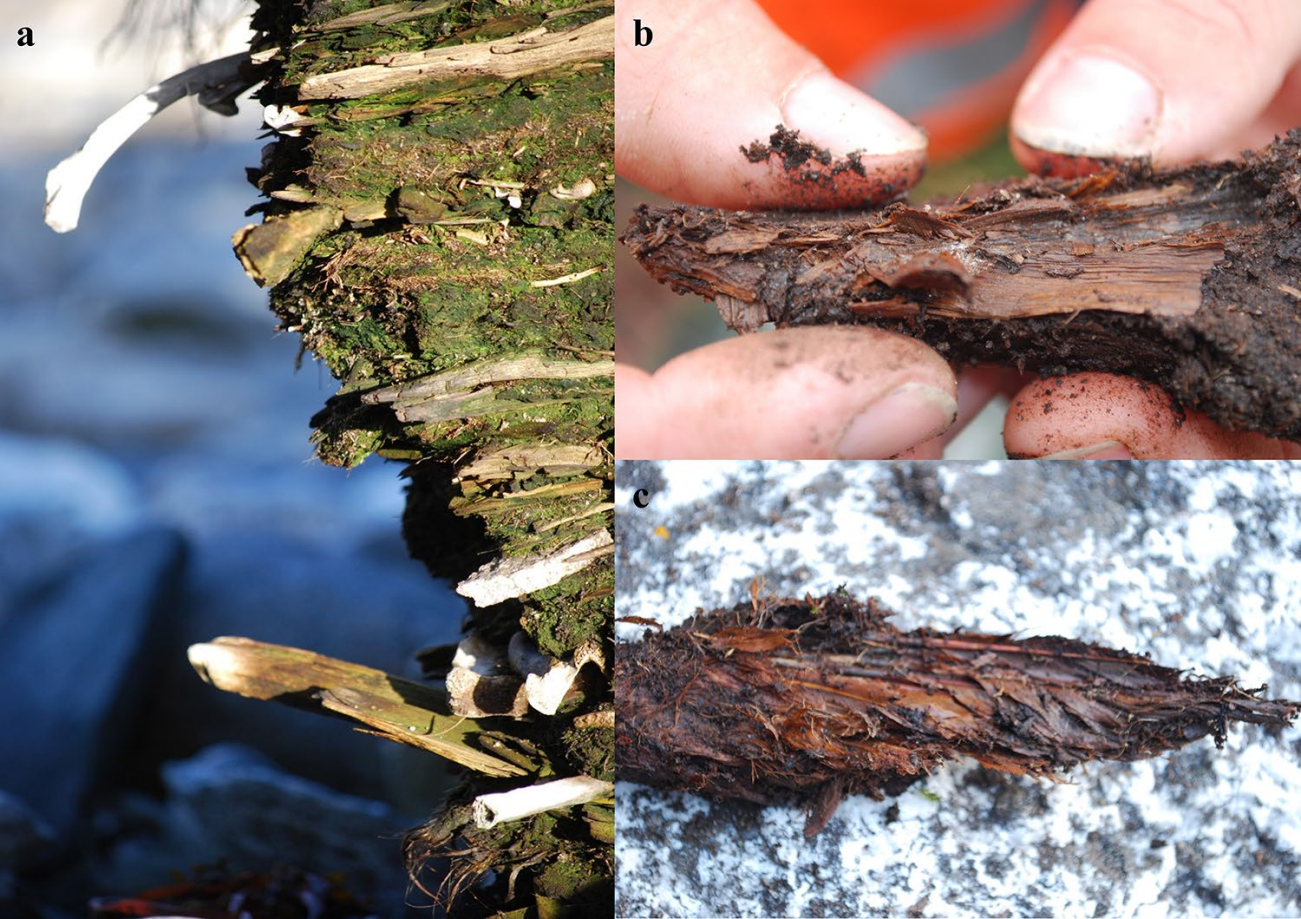
Archaeological middens are often remarkably well-preserved in Greenland. The midden at Kangeq (**a**), close to Nuuk in West Greenland, contains organic artefacts including animal bones, wood (**b**) and bird feathers (**c**). Most of the midden was accumulated by the Thule culture Inuits (1300 EC–present), but layers from the Saqqaq (2500–800 BC) and Dorset (300 BC–600 AD) cultures are also present (Photos: Jørgen Hollesen, National Museum of Denmark).

Greenland serves as a good example of what is at stake. Here archaeological sites represent an irreplaceable record of exceptionally well-preserved material remains spanning over 4000 years of human history. Individuals from past eras, whether they be Paleo-Inuit, Norse, Thule culture Inuit, or European, share a common trait in that they all exhibited a propensity to discard their domestic refuse near the front entrances of their dwellings. This practice resulted in the creation of trash heaps or middens (Fig. 1). These middens, which are often remarkably well-preserved, have proven to be a scientific boon for archaeologists conducting research in Greenland^10–14^.

Recovered artefacts and organic remains from Greenlandic middens have inspired great excitement. However, this excitement goes hand in hand with concern, as more and more evidence suggest that climate change may accelerate the decay of middens and their organic contents^5,7–9,15^. Recently it has been predicted that the majority of organic archaeological deposits in the Nuuk fjord area could lose their scientific value within the next 80 years, with the most rapid degradation expected to occur in the continental inland areas of the region, where the remains from the Norse Western Settlement (Vestribygð) are located^6,16^.

In South Greenland, approximately 500 km south of the Nuuk fjord, archaeologists are also facing a reality where the recovery of well-preserved organic materials is becoming increasingly scarce. Over the past two decades, archaeologists working in South Greenland have become acutely aware of the rapid and complete loss of previously outstanding organic preservation on a large proportion of Norse farms of the Eastern Settlement. Coring surveys carried out by researchers from the Greenland National Museum in South Greenland have revealed that the majority of sites seem to be in a state of advanced or complete degradation. To date, no studies have been published to track this escalating decay, and there is no direct evidence to corroborate its attribution to recent climatic change. Fundamentally, it is very difficult to document and quantify previous or ongoing microbial degradation of buried archaeological deposits. For example, at sites without prior or poor excavation records, it is challenging to verify whether decay is occurring presently or has occurred at various intervals in the past. Additionally, archaeological sites and materials are often heterogeneous and thus it is difficult to determine whether discrepancies between the current state of preservation and that observed in the past stem from climate change or other external factors, such as local terrain and depositional variations or material differences. Occasionally, comparison with historic data or samples from earlier excavations is possible^7,17^. However, such comparisons require substantial baseline data and necessitate a statistical approach involving the description and comparison of many old and new samples^18^. Unfortunately, such baseline data is often unavailable in Greenland.

This study is a first attempt to quantify the potential effects of recent climate change (1983–2022) on organic archaeological deposits within the UNESCO World Heritage site Kujataa in South Greenland. This is done based on comprehensive on-site investigations and analyses of local and regional climatic conditions. Furthermore, we use a numerical model approach to assess the net effect of recent changes in air temperature and precipitation on preservation conditions within archaeological deposits at four well-known sites in the area. As discussed above, we lack detailed information on the state of preservation as it may have existed before. Therefore, we use our model to answer the following basic question: *What would happen to “well-preserved” organic archaeological deposits if subjected to the diverse meteorological conditions prevalent in our study area over the past 40 years?* Based on results from previous degradation studies of organic archaeological materials^5,7,9^, we focus on the oxic degradation of organic deposits that contain important residues of human subsistence and in which various types of artefacts and ecofacts are embedded.

### Study region and study sites

**Figure 2:**
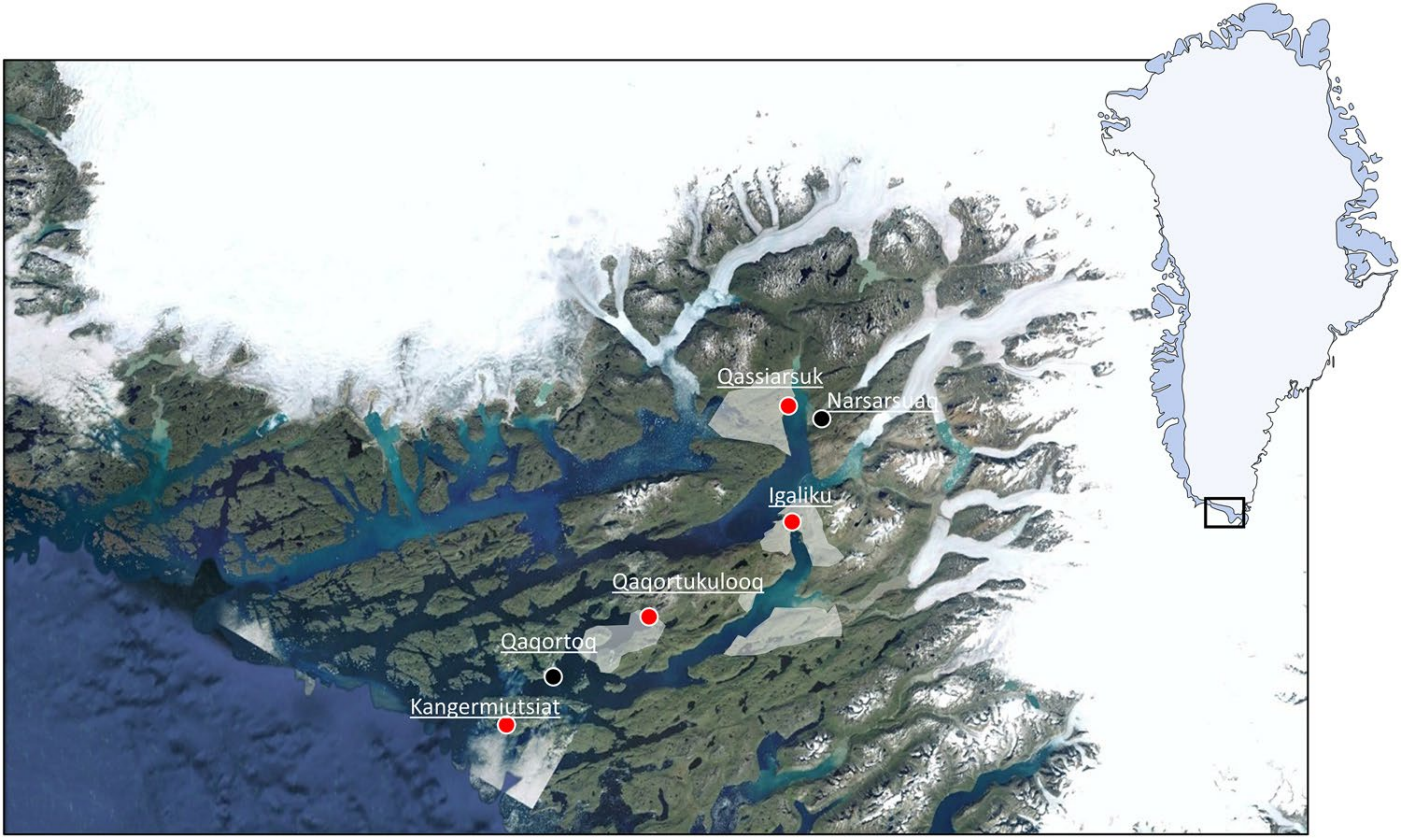
Study site locations. The four study sites (red dots) are located in or close to the component parts of the UNESCO World Heritage area Kujataa in South Greenland (grey areas). Automated monitoring stations were installed at the sites. In addition, data from two official meteorological stations was used (black dots). Figure was generated by Jørgen Hollesen and is based on a Landsat satellite image obtained with Google Earth Pro v7.3.6 (https://earth.google.com/).

The UNESCO World Heritage site of Kujataa, situated in the Kujalleq municipality of South Greenland (Fig. 2), is renowned as a medieval Norse and later Inuit subarctic agricultural landscape. Kujataa comprises five component parts, collectively representing the demographic and administrative core of two farming cultures: a Greenlandic Norse agricultural community spanning from the late 10th to mid-15th century and a pastoral Inuit farming society from the 1780s to the present^19^.

From 2021 to 2023, field work was conducted at four archaeological sites in the area (Fig. 2 and Supplementary Table S1). The sites were selected to represent archaeological deposits that have been exposed to the diverse climatic conditions encountered in the study region (Supplementary Fig. S1). Three of the sites—Qaqortukulooq (NKAH 4427), Igaliku (NKAH 2252), and Qassiarsuk (NKAH 2230) are located within Kujataa and rank among some of the most significant Norse sites in South Greenland. The fourth site, Kangermiutsiat (NKAH 2116), lies outside the UNESCO area and exemplifies a typical Inuit winter settlement situated on the outer coast. At all sites, the archaeological deposits lie within the uppermost section of the subsoil, covered by a 5–10 cm thick upper organic topsoil layer of turf.

## Results

### Characteristics of the archaeological deposits

To evaluate the current state of preservation, small soil pits were excavated in midden deposits at each of the four study sites. At the three Norse sites, Qaqortukulooq, Igaliku, and Qassiarsuk, the state of preservation was extremely poor. No preserved organic artefacts were discovered, and only the very last remnants of bones could be identified as reddish-colored markings in the soil. At the fourth study site, the coastal Inuit site Kangermiutsiat, the state of preservation was slightly better, with the presence of wood, skin and bone artefacts. However, the wood and bone artefacts were in a poor state of preservation (Supplementary Table S1).

**Figure 3:**
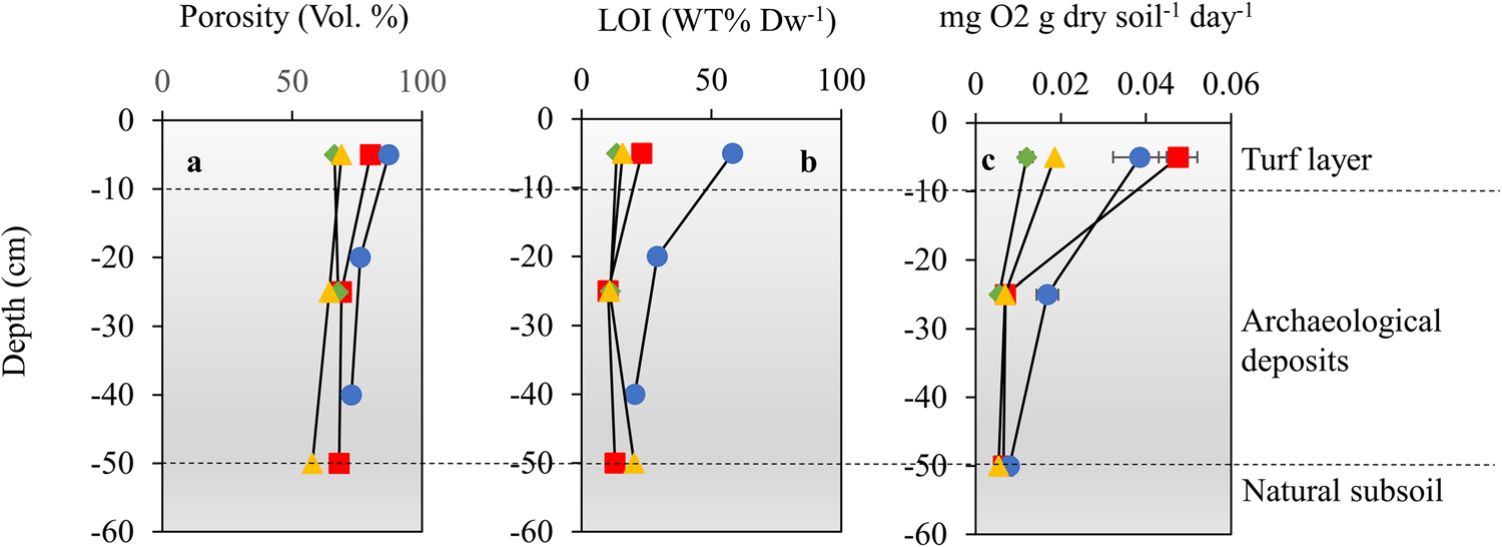
Characteristics of the archaeological deposits. Observed values of porosity (**a**), organic content (**b**) and oxygen consumption rates (**c**) at the sites Kangermiutsiat (blue circles), Qaqortukulooq (green diamonds), Igaliku (yellow triangles) and Qassiarsuk (red squares). Horozontal bars show ± 1 s.d.

Measurements of porosity, loss on ignition (LOI), and oxygen consumption were carried out on soil samples obtained from each of the soil pits (Fig. 3). In combination, these three parameters provide an indication of the quality of the organic content and its degradability. With relatively low porosities and LOI and very low oxygen consumption rates (Fig. 3), the results contribute further evidence that the organic archaeological deposits at the three Norse sites are highly degraded and in a very poor state of preservation. At Kangermiutsiat, the organic deposits have a higher organic content (Fig. 3b) and are more reactive (Fig. 3c) when compared to the other three sites.

### Environmental conditions at the study sites

**Figure 4:**
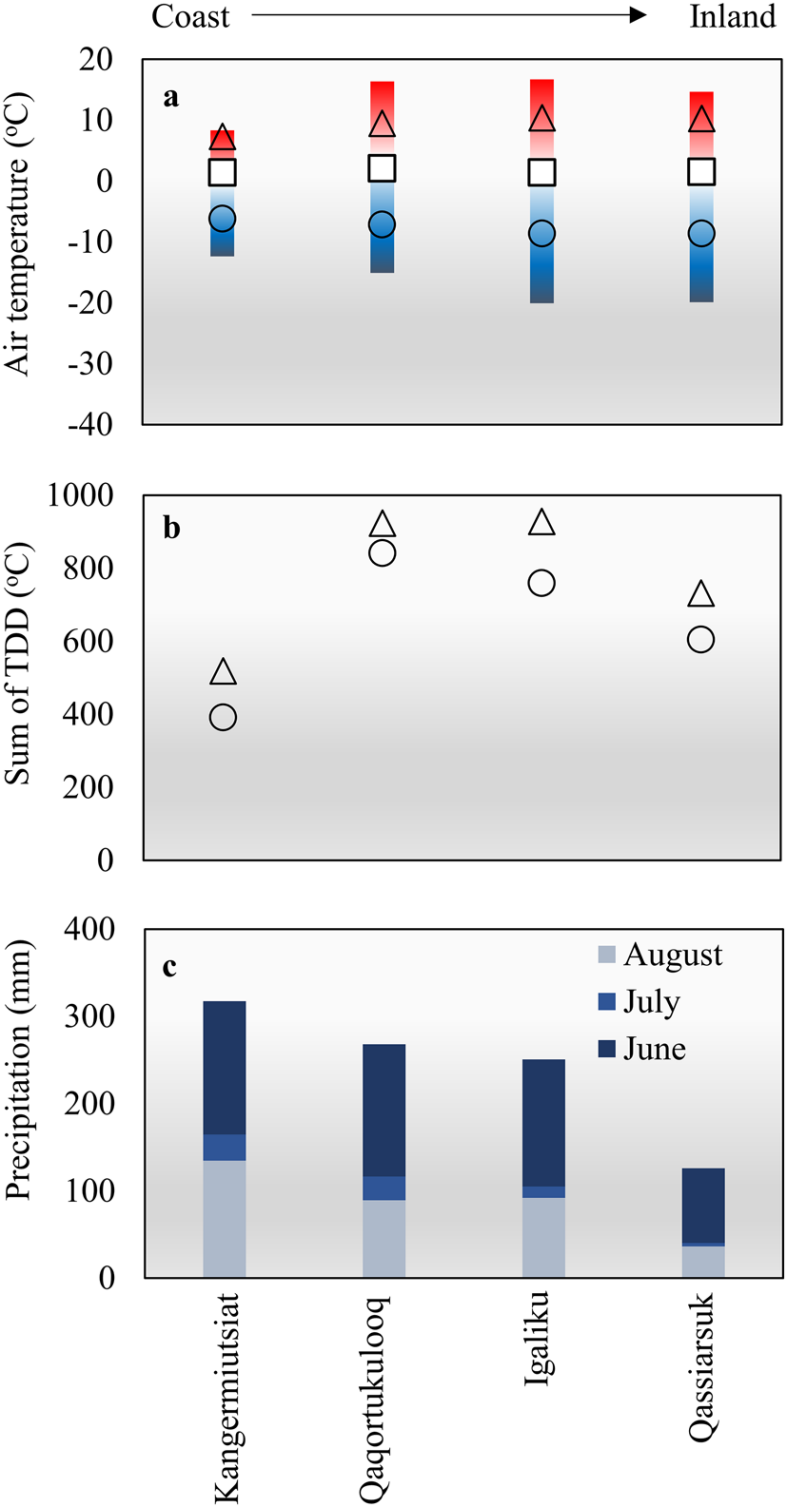
Meteorological conditions at the five study sites in 2022. (**a**) The mean air temperatures for the whole year (squares), the summer (triangles) and the winter (circles). The vertical color bars show the amplitude from the coldest to the warmest mean daily temperature registered during the year. (**b**) Sum of thawing degree days (TDD) for the summer period (June to August) in 0.25 m depth (triangles) and 0.50 m depth (circles). (**c**) The sum of precipitation during the summer period.

To investigate environmental conditions, automated monitoring stations were installed at each of the four study sites in August 2021 and measurements were conducted until July 2023. In 2022, the mean annual air temperature varied between 1.4 and 2.1 °C at the four study sites, with a noticeable rise in seasonal variation when going from the outer coast to the inner fjord (Fig. 4a). The mean summer temperature at the outer coast site, Kangermiutsiat (Fig. 4a), was 2–3 °C lower than at the other sites, with the maximum mean diurnal temperature reaching only 8.4 °C compared to between 14.6 and 16.7 °C at the three other sites. The overall trends in air temperature were also mirrored in soil temperatures. This is expressed in Fig. 4b where the average temperature for each day during summer has been added to calculate the sum of Thawing Degree Days (TDD) at depths of 0.25 m and 0.50 m. As can be seen in Fig. 4b the total amount of TDD is almost 50% lower at the outer coast compared to further inland.

We also observed a significant difference in the amount of rain falling during the frost-free period, with inland sites receiving substantially less rainfall than at the outer coast (Fig. 4c). Soil moisture contents were measured at the three inner fjord sites (Qaqortukulooq, Igaliku, and Qassiarsuk). With the percentage of water-filled pore spaces ranging between 40 and 80% during the summer period, the measurements indicate that soil moisture contents in the upper 0.5 m of the soils were well below water saturation at all three sites (Supplementary Fig. S2).

### Climatic changes from 1980 to 2023

To analyze variations in air temperatures and precipitation from 1980 to 2023, we employed data from Danish Meteorological Institute’s (DMI) official weather stations in Qaqortoq (outer coast) and Narsarsuaq (inner fjord) (Fig. 2)^20^. Prior to this, we evaluated the compatibility between our own observations at each study site and observations from the nearest of the two weather stations, and in all cases discovered highly significant correlations (Supplementary Figs. S3 and S4).

The data from the two meteorological stations show that the mean annual air temperature has risen by 1.5 °C at the outer coast and 1.7 °C in the inner fjord when comparing the means for 1981–2000 and 2001–2020 (Supplementary Fig. S4). The most significant increase occurred during the winter, with 2.4 °C in Qaqortoq and 2.9 °C in Narsarsuaq, while summer temperatures have increased by 0.9 °C in both areas.

A noticeable decline in precipitation rates has also been observed during the same period. In Qaqortoq, the mean for 1981–2000 was 977 mm compared to 863 mm for the period 2001–2020, and in Narsarsuaq, it was 665 mm compared to 557 mm (Supplementary Fig. S5). Precipitation rates have also decreased during the summer period at both locations. Particularly in Narsarsuaq, in the inner fjord, where the total precipitation from June to August decreased from 199 to 145 mm (27%).

### Representativeness of study sites

**Figure 5:**
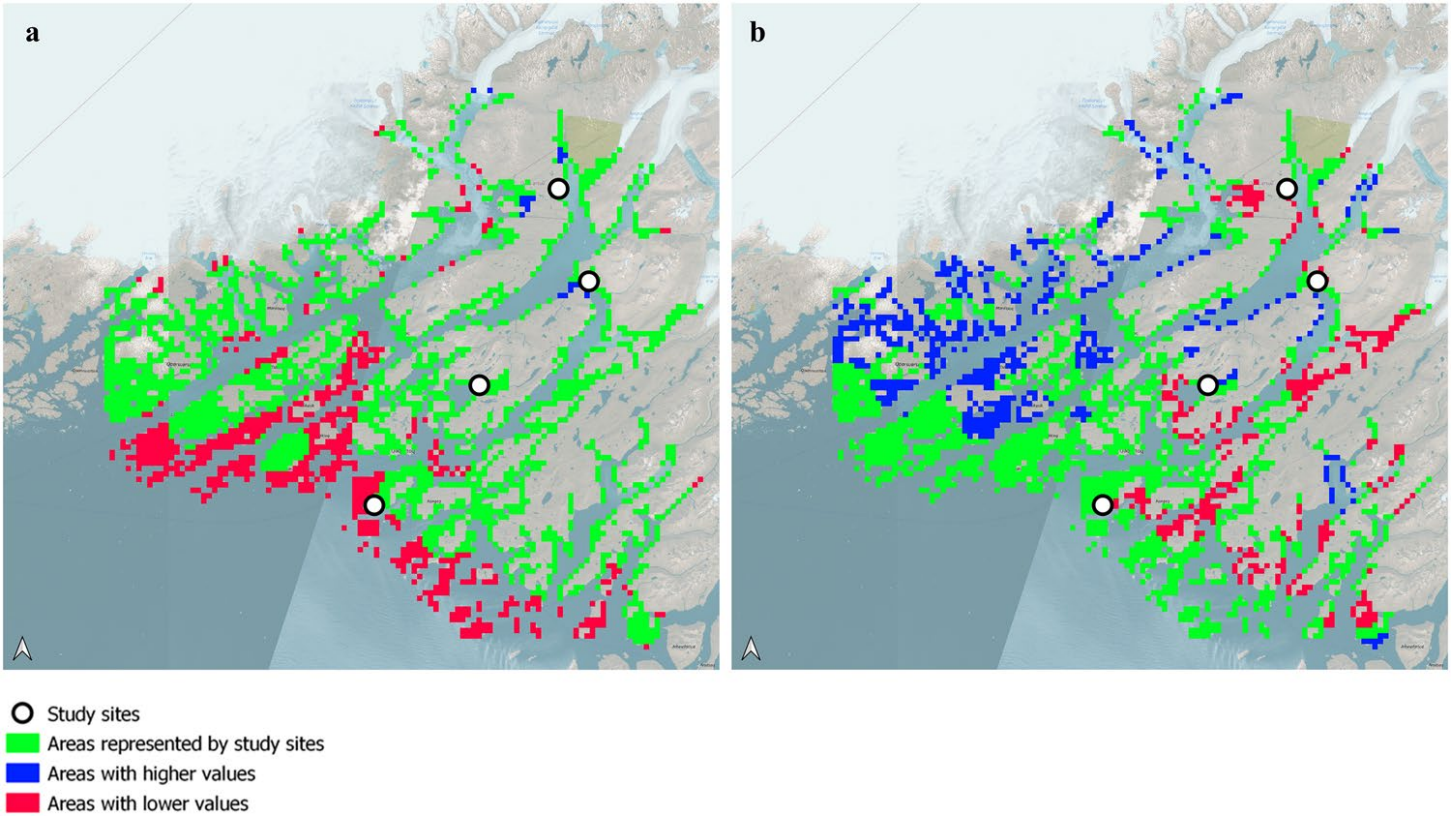
Representativeness of the four study sites in relation to: (**a**) mean air temperatures for the summer (June, July and August) from 2001 to 2020 and (**b**) mean sums of precipitation during the summer period from 2001 to 2020.

The representativeness of the study sites in terms of mean annual and mean summer air temperatures and sum of precipitation was assessed for the period 2001–2020 using data from the regional model HIRHAM5 at a horizontal resolution of 0.44° × 0.44°, driven by the global coupled model EC-Earth3^21^ (Fig. 5 and Supplementary Fig. S6) in a CMIP6 setup^22^. The mean summer temperatures at the four study sites represented 72% of the ice-free land below 100 m.a.s.l., where 85% of the archaeological sites in the area are located (Supplementary Fig. S7). In relation to the sum of precipitation during the summer, the four study sites represented 63% of the area. Of the areas that were not represented by our study sites 96% were colder, 4% warmer, 28% drier and 62% wetter.

### Future climate projections

HIRHAM5 was also run for future conditions following the SSP5-8.5 scenario which is a high-emissions scenario suggesting the likely outcome if society does not make concerted efforts to cut greenhouse gas emissions^23^. The results demonstrate that both mean annual and mean summer air temperatures at our study sites could increase by more than 4 °C by the end of the twenty-first century (Supplementary Figs. S8 and S9). At the same time the precipitation rates are projected to increase with 10–20% at three out of the four sites, however, at Qaqortukulooq a minor decrease in precipitation rates is projected during the summer.

### Modelling current conditions within the archaeological deposits

Soil temperatures, soil moisture contents, and the degradation of organic carbon (OC) over the past four decades were simulated at each of the study sites using the well-established heat-and-water flow model, the CoupModel^24^. This model has been previously used for similar purposes in other regions of Greenland^6,15,25^. In this study, the model configuration was based on experiences from previous investigations in the Nuuk area^6^, with some modifications implemented to tailor the model to the specific conditions of the new study sites (see “Methods” section).

**Figure 6:**
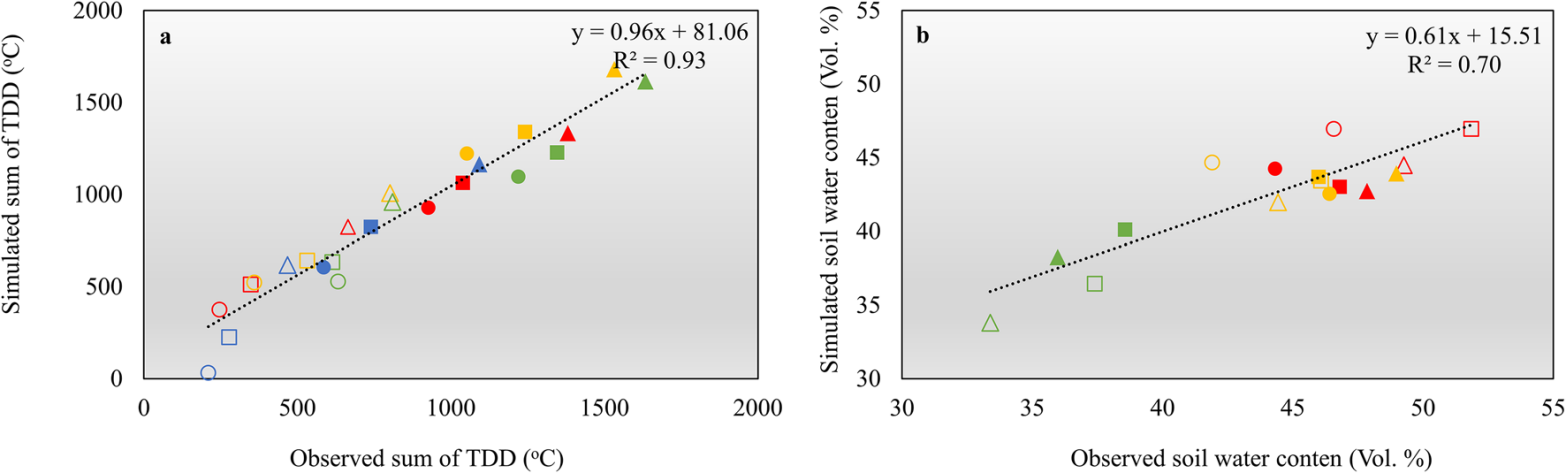
Linear regressions between simulated and observed data for the sites Kangermiutsiat (blue), Qaqortukulooq (green), Igaliku (yellow) and Qassiarsuk (red. (**a**) Observed versus modelled sum of thawing degree days (TDD). (**b**) mean simulated and mean observed soil water contents. Results are shown for 0.05 m depth (triangles), 0.25 m depth (squares) and 0.50 m depth (circles). Filled symbols represent values for the frost-free period during the calibration period from 1. January 2022 to 31. December 2022 and open symbols for the frost-free period during the test period from 1. January 2023 to 30. July 2023.

The model was first calibrated for each site based on one year of measured soil temperatures and water contents (January 1 to December 31, 2022) (Supplementary Figs. S10 and S11). Subsequently, the model was tested using measurements of soil temperatures and water contents from January 1, 2023 to July 30, 2023. High R-squared values (0.35 to 0.95) and low mean differences (− 1.27 to 0.83 °C) for both the calibration and test runs show that the model can accurately represent temperatures in the upper part of the soil where archaeological deposits are found (Supplementary Table S2). The largest discrepancy between simulated and observed temperatures occurs during the winter period, which can be attributed to uncertainties related to snow cover. However, when focusing solely on the frost-free period, when most microbial degradation occurs, the model performs very well. This is illustrated in Fig. 6a where observed and modeled TDD are compared.

With low mean errors (Supplementary Table S3) the model also performs well in terms of describing the absolute soil moisture content, especially during the frost-free period (Fig. 6b and Supplementary Fig.S11). However, as evident from the relatively low r-squared values, the model is less adept at capturing day-to-day variations in soil moisture content (Supplementary Table S3).

### Modelling recent changes

**Figure 7:**
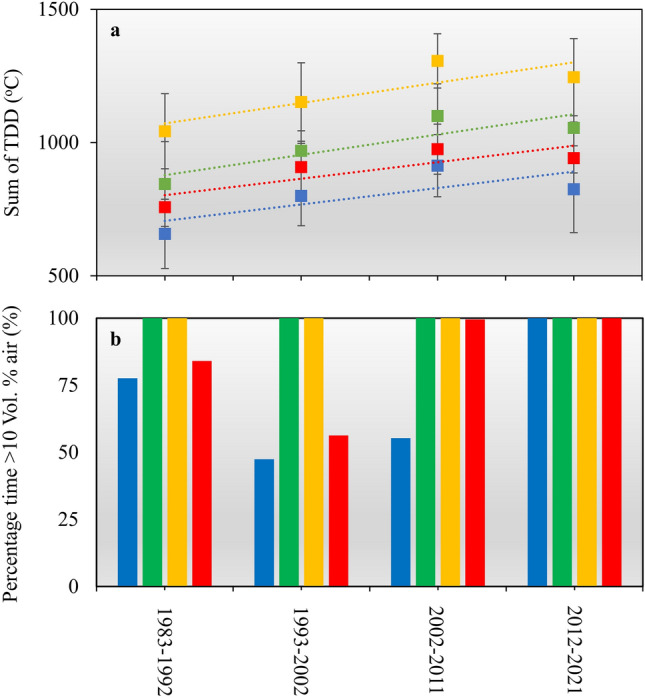
Modelled 10-year means of: (**a**) The sum of TDD during the summer period. (**b**) the percentage of time the soil moisture content is more than 10 Vol.% below saturation during July and August (100% = 62 days). The data represents the conditions in 0.25 m at Kangermiutsiat (blue), Qaqortukulooq (green), Igaliku (yellow) and Qassiarsuk (red). Vertical bars show ± 1 s.d.

The model setup was used to examine how changes in air temperatures and precipitation rates over the past 40 years have influenced soil temperatures and soil moisture contents at the four study sites. The findings demonstrate that recent warming has increased the amount of TDD by 128–201 °C at a depth of 0.25 m and by 133–202 °C at a depth of 0.5 m, when comparing the 1983–2000 and 2001–2020 means (Fig. 7a). Simultaneously, the duration of the frost-free period has expanded by 13–22 days at a depth of 0.25 m (Supplementary Fig. S12). The results also reveal that the diminishing amount of rainfall during the summer resulted in a reduction in soil moisture content. At all four sites, soil moisture content is currently more than 20% below saturation during the summertime (Fig. 7b and Supplementary Fig. S13).

**Figure 8:**
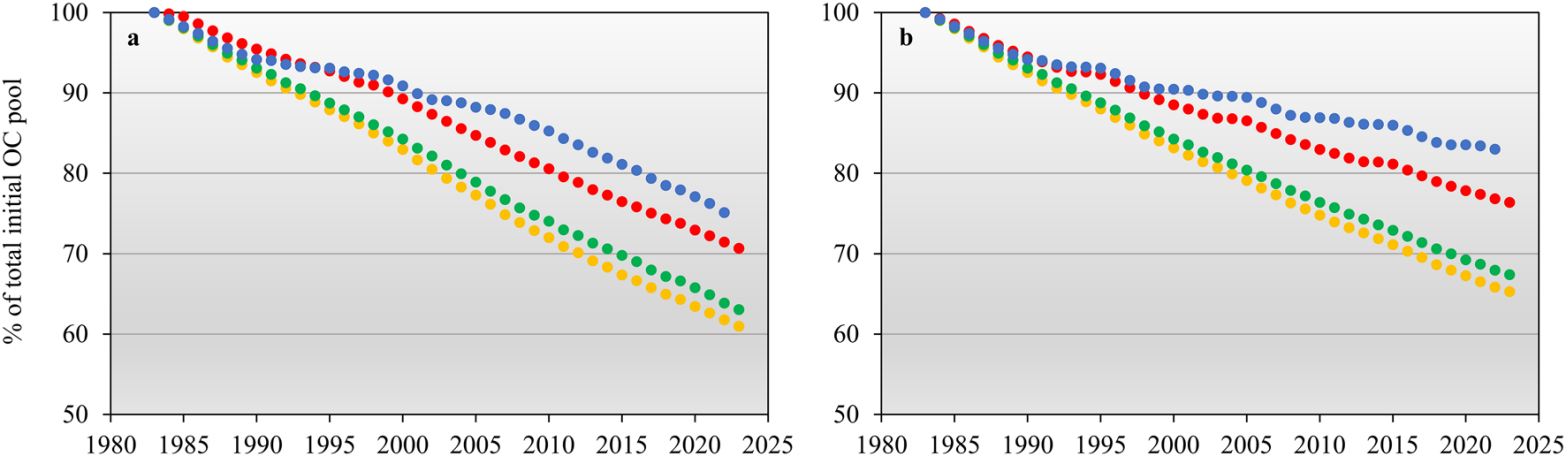
Modelled loss of OC from 1983 to 2022 in 0.25 m depth at Kangermiutsiat (blue), Qaqortukulooq (green), Igaliku (yellow) and Qassiarsuk (red). (**a**) Based on the meteorological condition observed from 1980 to 2023. (**b**) based on a "no climate change" scenario assuming that the meteorological conditions from 1983 to 1992 persisted until 2023.

We utilized a carbon degradation module within the CoupModel to investigate how the recent changes in soil temperatures and soil moisture content may have influenced the degradation of OC. Because of the poor state of organic preservation at three out of four of the study sites and the possibility that significant portions of the organic material may have already been degraded in 1983, the simulations were conducted based on the following question: *What would occur to well-preserved organic archaeological deposits if they were exposed to the varying climatic conditions found at our study sites?* The results reveal that 25–39% of the original slow OC pool (holding the archaeological OC fraction) in 0.25 m is lost between 1983 and 2022 (Fig. 8a). The most rapid degradation appears to occur in the middle part of the fjord system (Qaqortukulooq and Igaliku), whereas the degradation is noticeable lower at the outer coast (Kangermiutsiat) and in the very inner part of the fjord (Qassiarsuk). To quantify the net effect of recent changes in air temperature and precipitation, a "no climate change" scenario was also run in the model, assuming that the meteorological conditions from 1983 to 1992 persisted until 2023 (Fig. 8b). When comparing the two sets of model data the result show that the changes in climate from 1993 to 2023 potentially may have caused additional 4.3–8.3% of the total initial OC pool to decay which corresponds to 11.1–32.7% increase in the loss from 1983 to 2023. The largest net effect of recent changes in air temperature and precipitation is seen at Kangermiutsiat and Qassiarsuk.

## Discussion

We investigated four different archaeological sites situated along a 90-km transect extending from the western outer coast to the eastern inland ice sheet in South Greenland (Fig. 2 and Supplementary Fig. S1). Along this transect, we observed a pronounced difference in both the state of preservation and the climatic conditions (Figs. 3 and 4). At the coastal Inuit site of Kangermiutsiat, characterized by the lowest temperatures and the highest summer precipitation, the state of preservation was superior to that at the warmer and drier Norse sites located further inland. These results align with previous studies demonstrating that organic preservation is generally promoted by cold and very wet conditions^5,26^. However, it is important to note that Inuit and Norse deposits originate from different types of refuse and are thus not directly comparable. Moreover, as the Inuit deposits at Kangermiutsiat are later than those found at the Norse sites (Supplementary Table S1), the disparity in preservation status might also be age-related, meaning that Norse deposits have been exposed to decay for a much longer period.

Our analyses of local and regional climatic conditions reveal that air temperatures at our study sites have risen by 1.5–1.7 °C since 1983, a rate more than twice the pace of global annual temperature increase since 1981 (0.18 °C per decade)^27^. Although the most significant temperature increase has occurred during the winter months (2.4–2.9 °C), summer air temperatures have also risen by nearly 1.0 °C. Simultaneously, the overall amount of precipitation during the summer has declined by more than 25%. We employed the CoupModel to examine how these recent changes may have impacted soil temperatures and soil moisture content at our research sites. The findings demonstrate that the recent warming may have increased the quantity of TDD by 15–25% at both 0.25 m and 0.5 m depths (Fig. 7a), extending the duration of the frost-free period by 13–22 days, when comparing the 1983–2000 and 2001–2020 averages. (Supplementary Fig. S12). Additionally, the decrease in rainfall has resulted in a decline in soil moisture content during the summer season (Fig. 7b). Oxygen is a key controlling factor for the degradation of buried archaeological materials^28^. The concentration of oxygen is higher, and the supply of oxygen is much faster in air-filled soil pores compared to water-filled pores^29^. This is one of the primary reasons why draining of waterlogged archaeological deposits can lead to significant changes and rapid deterioration of organic materials^30^. Furthermore, fluctuating water levels and shifts between wet and dry conditions may also act to cause further degradation^31^. Past studies suggest that oxygen becomes accessible if the soil moisture content falls below saturation by more than 10–15%^28^. As shown in Fig. 7b and Supplementary Fig. S12, this threshold is consistently exceeded at all four sites during July and August at a depth of 0.25 m. For two of the sites (Qaqortukulooq and Igaliku), this has been the case throughout the entire period from 1983 to 2023, while for the other two sites (Kangermiutsiat and Qassiarsuk), conditions have gradually become drier.

The model was used to investigate how the changes in thermal and hydrological conditions may have affected the degradation of OC (Fig. 8). When assuming that the archaeological deposits at the four sites were equally well preserved in 1983, the model findings suggest that 25–39% of the archaeological OC at a depth of 0.25 m has been lost from 1983 to 2022. The spatial variation in the simulated loss of OC is closely linked to soil temperatures and soil moisture levels (Fig. 7). Therefore, the greatest simulated loss occurs at the two warmest and driest sites, Qaqortukulooq and Igaliku. In comparison to a scenario where meteorological conditions from 1983 to 1992 persisted until 2023, the results indicate that the recent changes in air temperature and precipitation may have enhanced the overall loss of OC from 1983 to 2023 by 11.1–32.7%. The model results suggest that recent changes might have had the most significant overall effect at the coastal site of Kangermiutsiat and at the most inland site of Qassiarsuk. At these two locations, the decrease in precipitation experienced from 2002 to 2021 has caused soil moisture to decrease and cross the threshold between anoxic and oxic conditions described earlier (Fig. 7b). For the other two sites (Qaqortukulooq and Igaliku), soil moisture levels are already below this threshold regardless of whether recent climate change is considered.

It is very difficult to determine at which point in time organic degradation has occurred. In the absence of a "preservation baseline" from 1983 for comparison, it is impossible to validate whether the poor preservation status observed at our Norse study sites stems from factors that emerged prior to or following 1983, possibly even centuries ago. Therefore, the results presented above should not be perceived as an attempt to simulate the actual site-specific degradation. Instead, they serve as a contribution to the broader assessment of significant climatic factors that we believe are very closely tied into the preservation potential in the region. These external climate driven pressures related to soil temperature and moisture content are not the sole determining factors but work in conjunction with a broad range of influences that include terrain type, soil quality, relative degree of annual solar exposure, overlying vegetation and other animal and human derived interferences.

Regardless of whether our study sites were well-preserved in 1983, the findings highlight that the current climatic conditions in Kujataa and South Greenland are not conducive to organic preservation. The model simulations suggest that the greatest risk of degradation lies within the relatively dry continental inland areas of the study region, where all Norse Viking Age settlements are situated. Consequently, the prospect of discovering well-preserved organic remains from the Norse appears to be diminishing, if not already lost. Furthermore, even at the "cold" and "wet" outer coast, the combined effects of rising summer temperatures and declining soil moisture levels may already be exerting a noticeable impact. As a result, if precipitation rates remain low in the coming years, archaeological deposits at coastal Inuit sites like Kangermiutsiat could also rapidly lose their scientific value.

In terms of temperature and precipitation, our study sites encompass most of the ice-free land below 100 m elevation (Fig. 5 and Supplementary Fig. S6), where 85% of the registered archaeological sites in the region are located (Supplementary Fig. S7). Based on this observation, we anticipate that the majority of archaeological sites in the region are exposed to climatic conditions that are comparable to those we have described. However, it is important to note that our model does not incorporate local factors such as topography (slope and aspect), which can significantly influence soil moisture content and the amount of incoming solar radiation^32^. Additionally, we have not included sites located at high altitudes or near glaciers or the inland ice. Consequently, there may be areas within Kujataa and South Greenland in general where the degradation of organic archaeological materials is expected to be less pronounced.

We employed the most up-to-date version of the EC-Earth3 and HIRHAM5 models and found that air temperatures within our study area under the SSP5-8.5 scenario could rise by more than 4 °C by the end of the twenty-first century (Supplementary Figs. S8 and S9). Additionally, precipitation rates are projected to increase by approximately 10–20% from 2017 to 2100. However, the timing and annual distribution of precipitation may also alter, resulting in longer dry spells during the summer and more intense rainfall events. Simultaneously, evapotranspiration and runoff are expected to rise, causing the amount of water that infiltrates into the ground to remain the same as today. As a result, the prospects for an improvement in preservation conditions appear bleak.

The CMIP6 ensemble consists of a large number of combinations of global and regional climate models. For Greenland, these models differ in the strength of the Atlantic meridional overturning circulation (AMOC), the near-surface part of which is commonly known as the Gulf Stream. The AMOC variability for present-day climate is well within the observational uncertainties, while future changes are found to be halfway between models with virtually no change and models that show a substantial decrease of the AMOC over the course of the twenty-first century^21^. So, depending on the model, preservation conditions could worsen slower, but also considerably faster than in the case of the model combination considered here.

## Conclusion

The designation of Kujataa as a World Heritage Site in 2018 is a significant recognition of South Greenland's unique and diverse geographic and cultural assets. However, it also presents a formidable challenge to conservation in a region undergoing rapid climate change. Over the past 40 years, the area has experienced a substantial warming trend, that when coupled with less precipitation, creates a dangerous combination with the potential to accelerate the degradation of organic materials, even at sites on the relatively "cold" and "wet" outer coast. Currently, there are 583 archaeological sites registered within Kujataa. Our findings underscore the possibility that the organic deposits at many of these sites may already be degraded and that we are facing a future where this problem will only intensify.

Within archaeology and cultural heritage management, the primary aim is to leave as much archaeological material as possible undisturbed in the ground, also known as in situ preservation^33^. One of the main justifications for in situ preservation of archaeology is to preserve cultural heritage for future generations of archaeologists who, with their improved methodologies and expanded knowledge, can extract greater value from the material. However, this approach becomes irrelevant if the cultural heritage is allowed to deteriorate to the point where the remains cease to exist. As exemplified in this study, it is challenging to detect ongoing microbial degradation of buried deposits, and as a result, we may lose valuable cultural assets without even realizing it. The sad reality is that in South Greenland, we will eventually have to come to terms with the irreversible loss of archaeological materials within our lifetime. However, in many other parts of Greenland and the Arctic, where the climate is still cold and wet, there is time to address the issue. But the longer we delay, these challenges will become more difficult to manage. Robust efforts for strategic salvage archaeology combined with a comprehensive inventory and conservation of the backlog of archaeological materials currently residing in labs and museums outside of Greenland could be one approach for compensating for this inevitable loss. Excavations in remote regions are, however, expensive and time consuming and thus we also need to consider alternate methods for obtaining and storing data. This necessitates exploring alternative methods for data acquisition and storage. Fortunately, studies have shown that DNA extracted from soil cores^34^ and small bone fragments^14^ can offer valuable insights into past cultures and their livelihoods. Therefore, soil samples and cores, even from degrading sites, can be a valuable resource for future studies, holding the key to unlocking a wealth of information about Greenland's past inhabitants.

Finally, we would stress that this process of change taking place above and below the ground is part of a larger narrative of dramatic shifting human relationships with the environment in the Anthropocene. The changes we are experiencing at this moment in time are important and can help inform and raise awareness around the consequences of the loss of heritage and what it will mean to future generations. While we may not be able to stop this process, through small interventions and policies of curated decay^35^ we can educate and empower local communities in their efforts to preserve and retain knowledge of the past, while speaking to the realities of an uncertain future.

## Methods

### Characteristics of the archaeological deposits

Soil pits were made at the study sites to investigate the state of preservation and to obtain soil samples for measuring the physical and chemical properties, as well as the degradability of the archaeological deposits. Measurements of porosity and loss on ignition were conducted on a total of 11 volume specific soil samples (100 cm^3^) taken in 0.05, 0.25 and 0.50 m depth at each of the four sites. The degradation potential of the archaeological deposits was investigated using the oxygen consumption method^36^. Measurements were made on triplicates from the 11 depth-specific samples from the four sites at 5 °C. The collected soil bulk samples were homogenized manually (stones, larger bones, and wooden fragments were first removed) and triplicates were extracted and used for measurements of O_2_ consumption. The triplicates were placed in vials, which were sealed using a disc of transparent plastic commercial oxygen barrier film (EscalTM), a silicone gasket and a screw cap with an aperture. Oxygen sensor foil (SF-PSt5-1223-01, PreSens) was glued to the inside of the oxygen barrier film, and the OxoDish® readers were placed on top of the vials. The decrease of headspace O_2_ concentrations with time was measured for each sample using SDR SensorDish® readers (PreSens Precision Sensing GmbH, Regensburg, Germany).

### Investigating environmental and present meteorological conditions

To investigate environmental conditions, automated monitoring stations were installed in August 2021 at each study site. Measurements were carried until July 2023. An overview of those environmental parameters monitored, and the equipment used is given in Supplementary Table S5. Furthermore, we used data from two official weather stations in Qaqortoq and Narsarsuaq^20^ to analyze variations in air temperatures and precipitation from 1980 to 2023 and as input in our numerical model.

### Modelling of site-specific conditions

In Hollesen et al.^6^, a new model set-up was developed in the one-dimensional numerical heat and water flow model, the CoupModel^24^, and used to simulate the effect of future climate change on the degradation of OC in similar types of archaeological deposits as studied here. We used the model configuration from this study with some modifications implemented to tailor the model to the specific conditions of the new study sites.

The model employed a 5.0-m-deep profile divided into 64 layers. The upper 0.5 m (11 layers) were considered to represent organic soil. The soil below 0.5 m was assumed to resemble the entisol/cryosols typically found in this region of Greenland. Surface temperature was calculated using an equation proposed by Brunt^24^ based on the air temperature. When the ground was snow-covered, surface temperature was adjusted using a weighting factor that took into account the thermal conductivities of the upper soil layer and snow density, along with the thickness of each layer. The model assumed that precipitation consisted solely of snow when air temperatures were below 0.5 °C. Water uptake by plants and soil evaporation were treated as a single flow from the uppermost soil layers. The distribution of water uptake among soil layers was determined using measured depth-specific root distribution, default values for potential transpiration rates (4 mm/day), and a growing season of 120 days. Water retention capacities and hydraulic conductivities were estimated from the measured porosity and organic content using the Mualem and Brooks & Corey equation^24^. The heat capacity (hc) and thermal conductivity (kh) were calculated as functions of soil solids and soil moisture. For unfrozen conditions, values were calibrated for each layer based on measured values from similar types of archaeological deposits^6^. For frozen conditions, default values for organic soils were employed. For layers beneath the archaeological deposits, values of *hc* and *kh* were based on default values for mineral soils.

Mean daily air temperatures, relative humidity, and precipitation rates were employed as meteorological input to the model. From January 1, 1980, to August 31, 2021, we utilized data from the nearest of the two official weather stations in Qaqortoq and Narsarsuaq. From September 1, 2021, to July 31, 2023, site-specific data collected via our monitoring stations were employed. The simulations commenced on January 1, 1980. However, to preclude any initial instability from affecting soil temperatures and soil moisture contents, the initial three years of simulations were regarded as a "warm-up" period and were not used. The model configuration was then employed to simulate the environmental conditions at the sites from 1983 to 2022 to identify changes in the thermal and hydrological conditions within the archaeological layers.

We also used a carbon degradation module in the CoupModel to investigate potential consequences of recent climatic changes on the degradation of OC. The module is described in detail in Hollesen et al.^6^ but the overall concept is: The total pool of OC is divided into three subpools; a fast (5%), a slow (50%) and a passive pool (45%). The turnover times for each of the pools (under drained conditions at 5 °C) corresponded to 1, 50 and > 4000 years respectively. The archaeological fraction of OC is considered to be in the slow pool. The absolute loss of carbon is calculated for each soil layer and adjusted to the simulated depth-specific temperatures and soil moisture assuming that the degradation rate is limited by high and very low water contents.

### Climate projections

To obtain a regional overview of recent and expected future variation in temperature and precipitation in the area we used the HIRHAM5 driven with the EC-Earth3 model^21^. The model was run for two periods: 1971–2014 and 2015–2100, representing present‐day and future conditions following the SSP5-8.5 scenarios. SSP5-8.5 is a high emissions scenario with a global warming of 4.4 °C^23^. We used the data to calculate yearly and summer means/sums of air temperature and precipitation for the periods 2001–2020 and 2081–2100 in order investigate the recent and expected future spatial variation of these two parameters. HIRHAM5 data has a resolution of appr. 5.5 × 5.5 km^2^. However, we interpolated the data to 1 × 1 km^2^ resolution using ArcGIS Spline tool with tension setting of 5 (weight) and 8 (points).

The raster cells from the HIRHAM5 interpolated dataset of temperature and precipitation was analyzed to determine number of cells represented by the study site cells. Here we only focused on land areas that are below 100 m.a.s.l which were selected according to the Digital Height Model of Greenland^37^. A polygon was established delineating all land between 0 and 100 m.a.s.l. in the area. Then an overlay analysis was conducted, selecting all interpolated HIRHAM5 cells with > 50% of cell area within the polygon. HIRHAM5 cell values were sampled for the four study sites. For both air temperature and precipitation, the lowest and highest values sampled were set to define the lower and upper boundaries for a range search where the number of interpolated HIRHAM5 cells with values within the range were counted.

To assess the representativeness of our study sites to sites in relation to the total number of archaeological sites in the study area, we used spatial data from the Greenland national database of cultural heritage monuments and properties ‘Nunniffit’. The study sites were set to represent other sites located below 100 m.a.s.l. With the polygon of land below 100 m.a.s.l. an overlay analysis was conducted to classify sites located below 100 m.a.s.l. The number of sites located below 100 m.a.s.l. was then compared to the total number of sites in the area.

### Supplementary Information

Supplementary Information.

## Data Availability

The datasets generated during and/or analysed during the current study are available from the corresponding author on reasonable request.
