# Supplementary material for: Assessing the consequences of recent climate change on World Heritage sites in South Greenland
Source: Sci Rep. 2024 Apr 28;14:9732. doi: 10.1038/s41598-024-60397-9 (PMC11056380; doi:10.1038/s41598-024-60397-9)
Supplement: Supplementary file 1 — Supplementary Information. [file 41598_2024_60397_MOESM1_ESM.pdf]

Supplementary information:

Assessing the consequences of recent climate change on World Heritage sites in South Greenland

Jørgen Hollesen[1*], Malte Skov Jepsen[1], Martin Stendel[2] & Hans Harmsen[3]

[1] *Environmental Archaeology and Materials Science, The National Museum of Denmark, IC Modewegsvej, Brede, DK-2800 Lyngby, Denmark*

[2] *Danish Meteorological Institute (DMI), Sankt Kjelds Plads 11, DK-2100 Copenhagen Ø Denmark*

[3] *Greenland National Museum & Archives, Hans Egedesvej 8, Boks 145, 3900 Nuuk, Greenland*

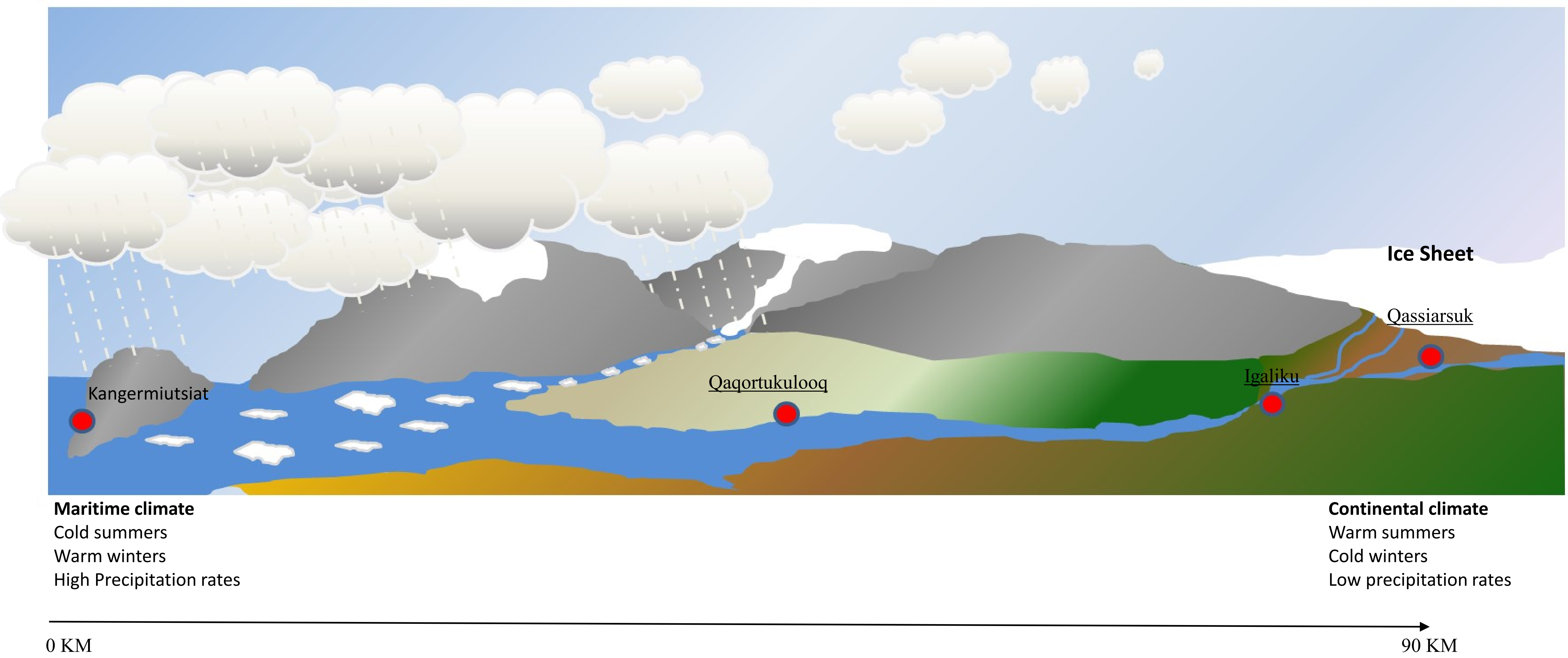

Supplementary Fig. S1: Conceptual illustration of the site selection strategy used in this study. The four study sites are located along a climate gradient stretching from the outer coast to the inner fjord and represent a suite of different climatic and environmental conditions. Illustration made by Jørgen Hollesen.

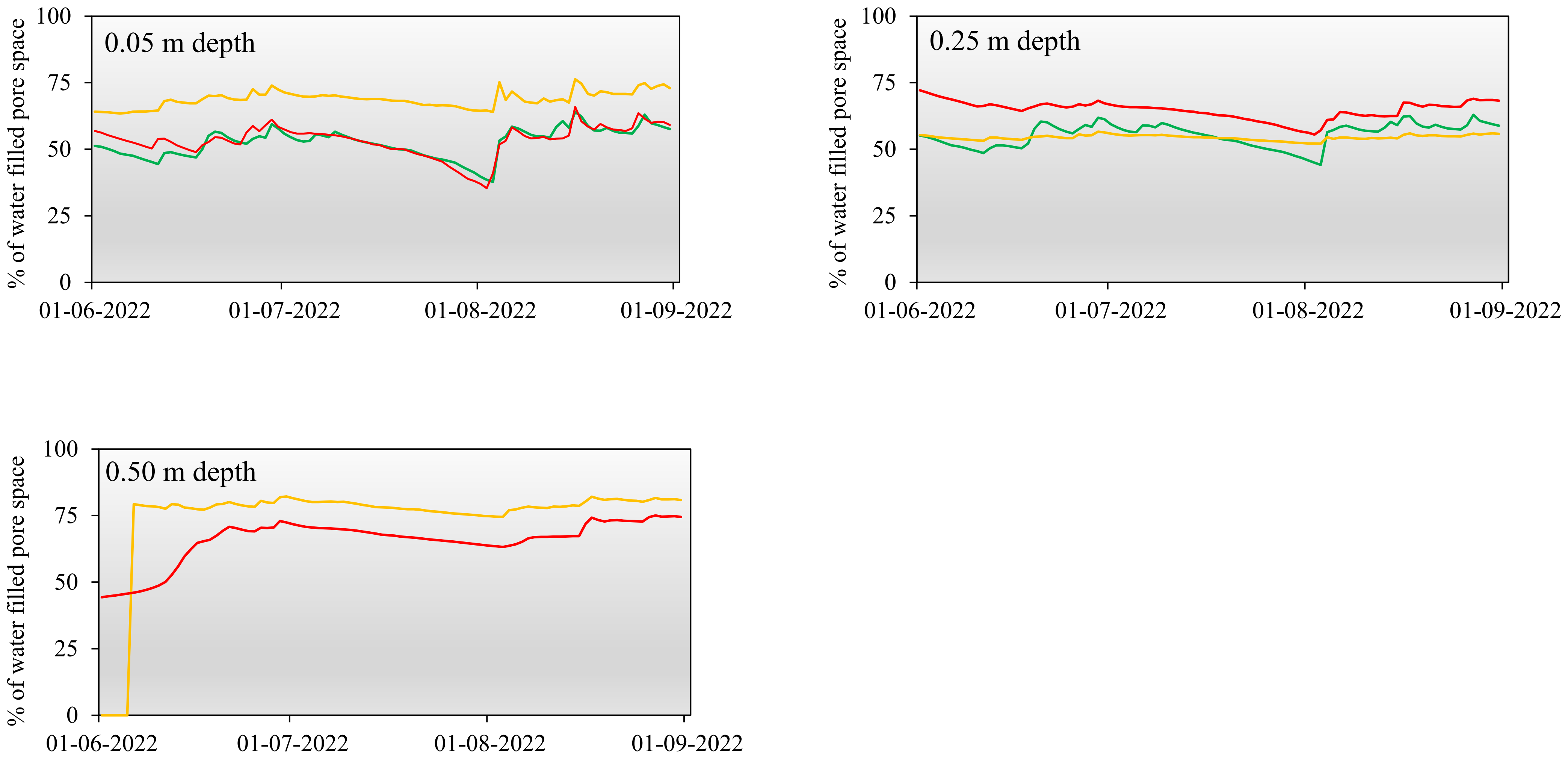

Supplementary Fig. S2: Percentage of water filled pore space during the summer period 2022 in 0.05, 0.25 and 0.50 m depth at Qaqortukulooq (green), Igaliku (yellow) and Qassiarsuk (red).

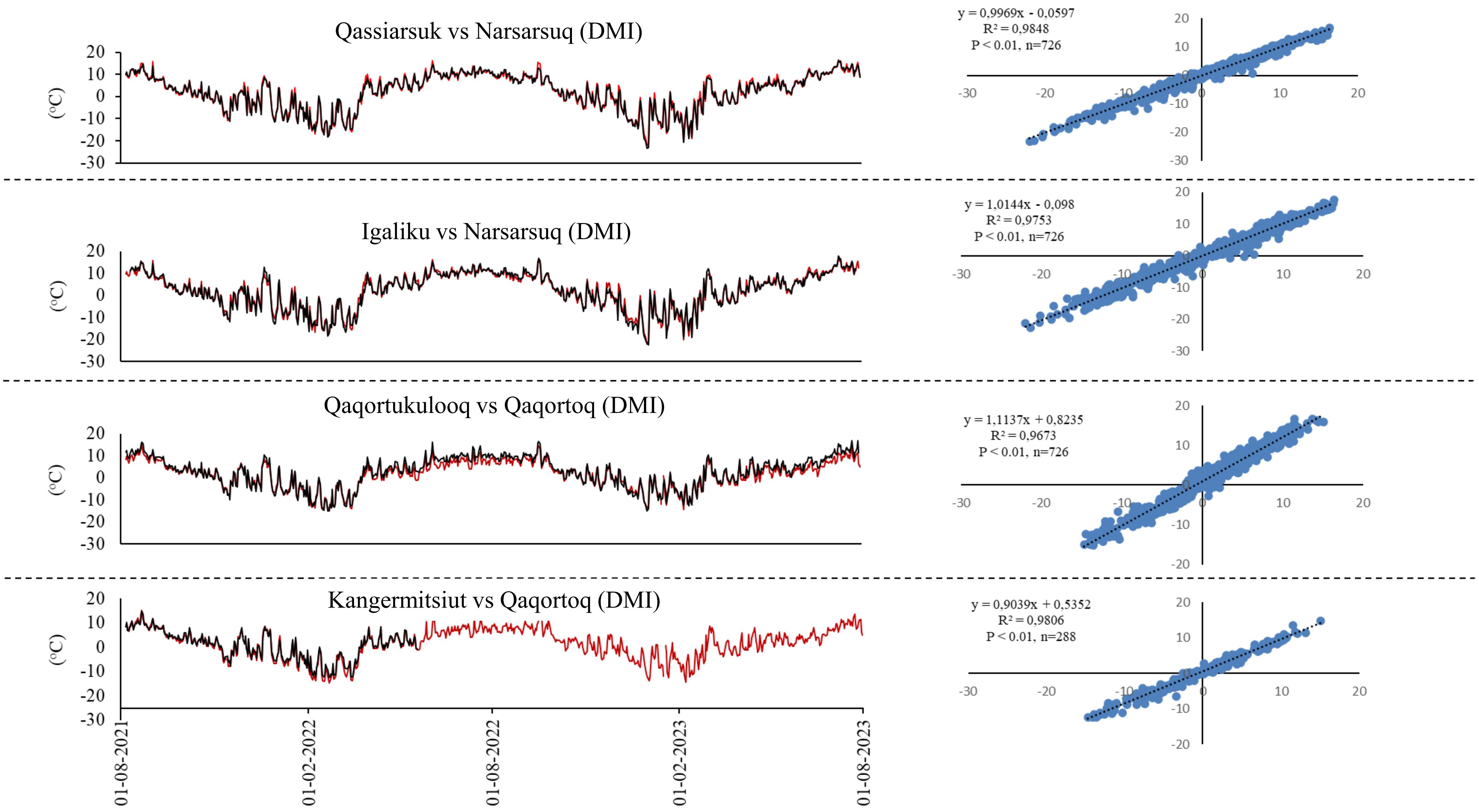

Supplementary Fig. S3: Left: Air temperatures observed at the four study sites (black) versus air temperatures measured at two official meteorological stations (red). **Right:** Linear regression between observations from the official meteorological stations (x) and the site-specific observations (y).

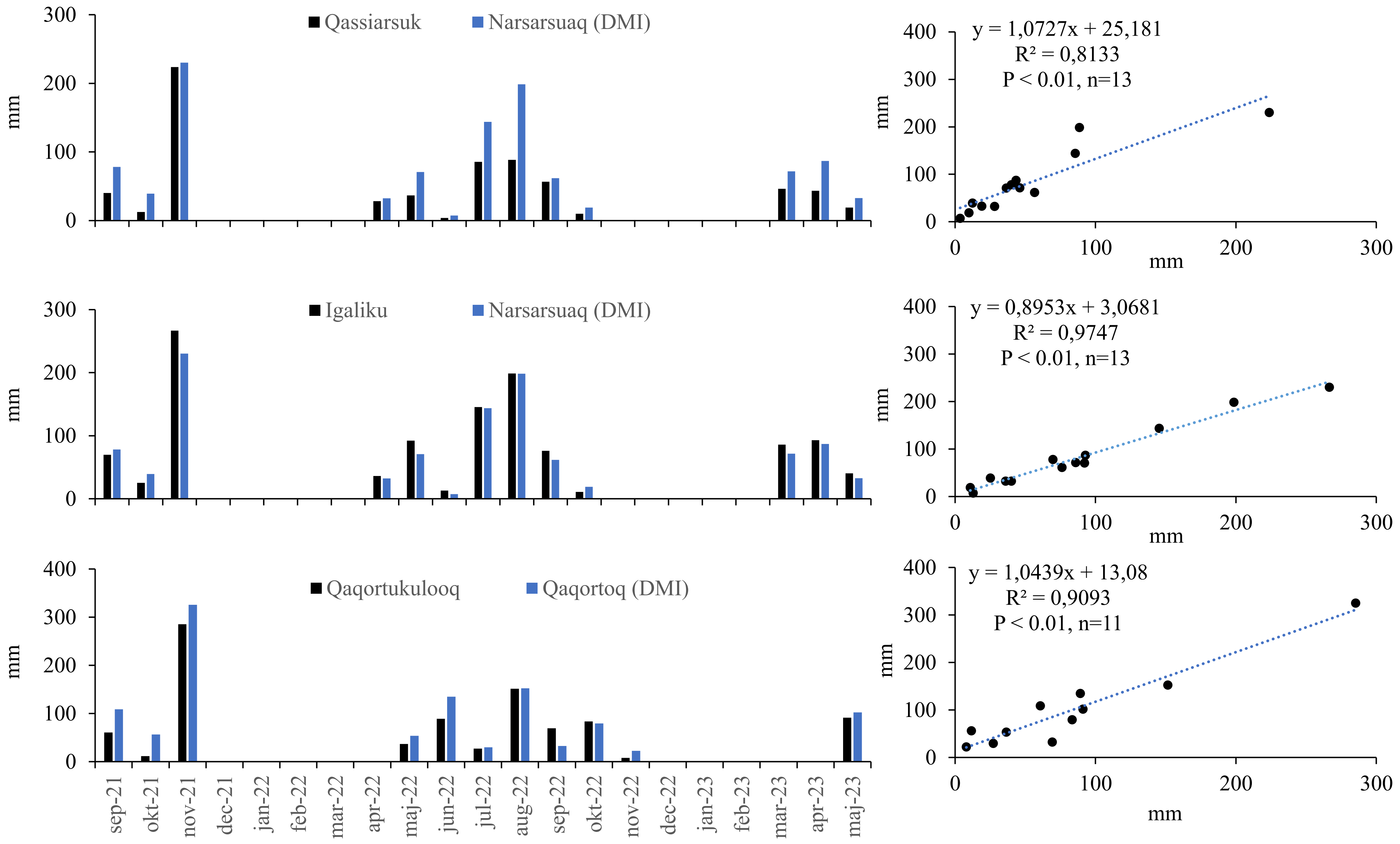

Supplementary Fig. S4: Left: Monthly sum of rain observed at the four study sites (black) versus rain measured at two official meteorological stations (red).

Right: Linear regression between the site-specific observations (x) and observations from the official meteorological stations (y).

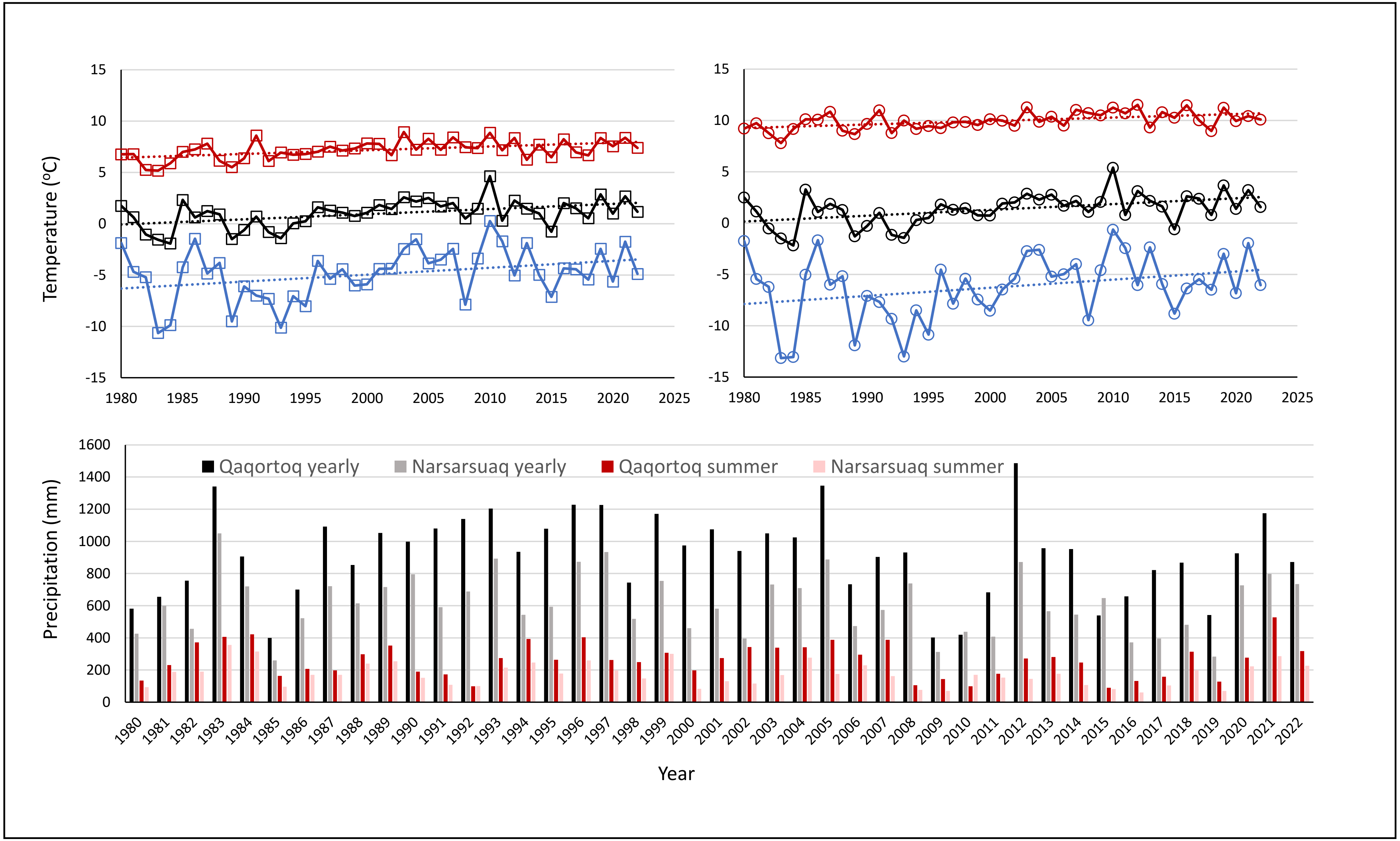

Supplementary Fig. S5: Upper: Mean annual (black), summer (red) and winter (blue) air temperatures in Qaqortoq (left) and Narsarsuaq (right) from 1980-2022. Lower: Precipitaion rates from 1980-2022.

Supplementary Fig. S6: Representativeness of the four study sites in relation to: (a) mean annual air temperatures; (b) mean air temperatures for the summer (1. June to 30. August); (c) mean annual sums of precipitation and; (d) sums of precipitation during the summer period. The data is based on means for the period from 2001-2020 .

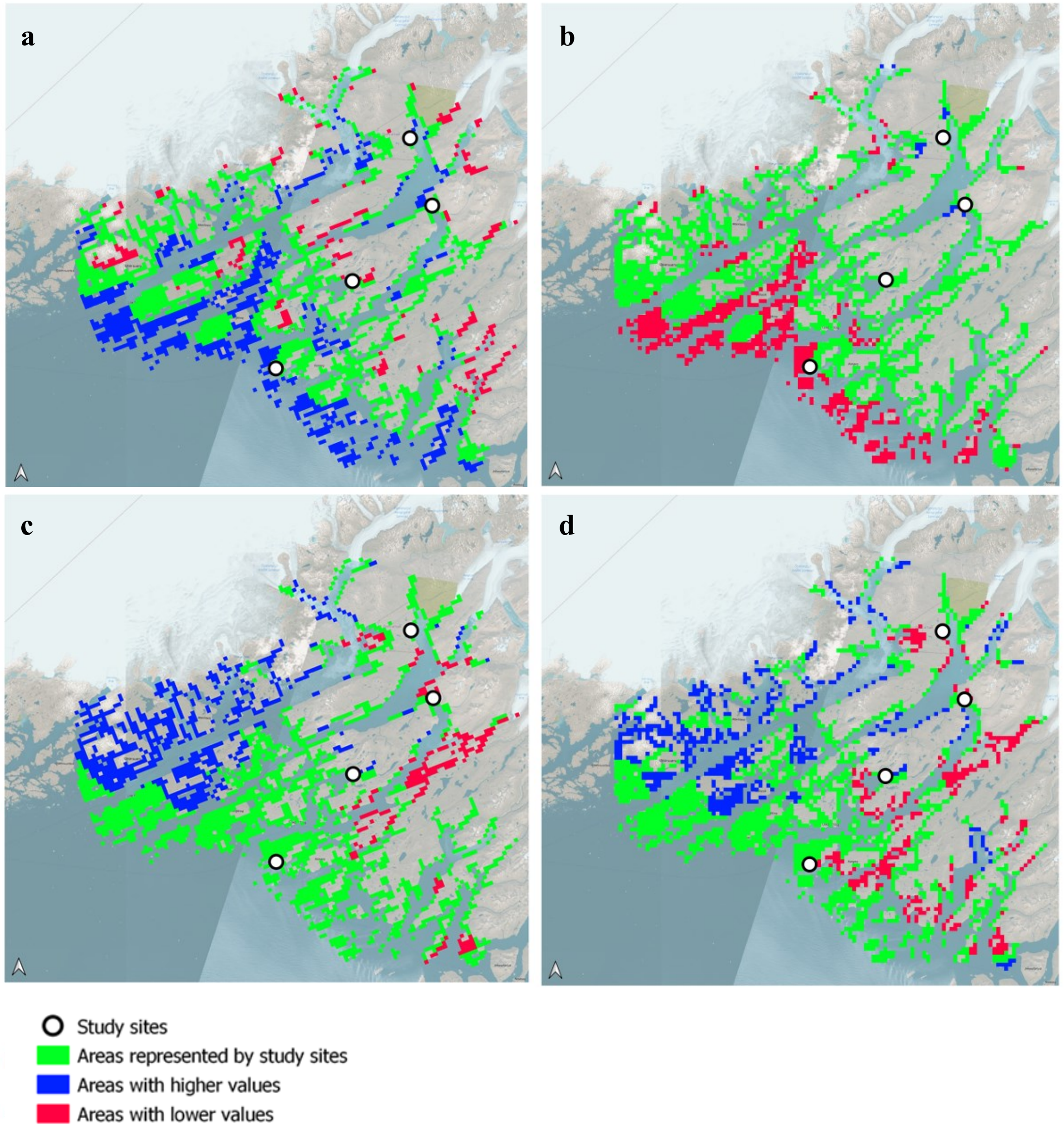

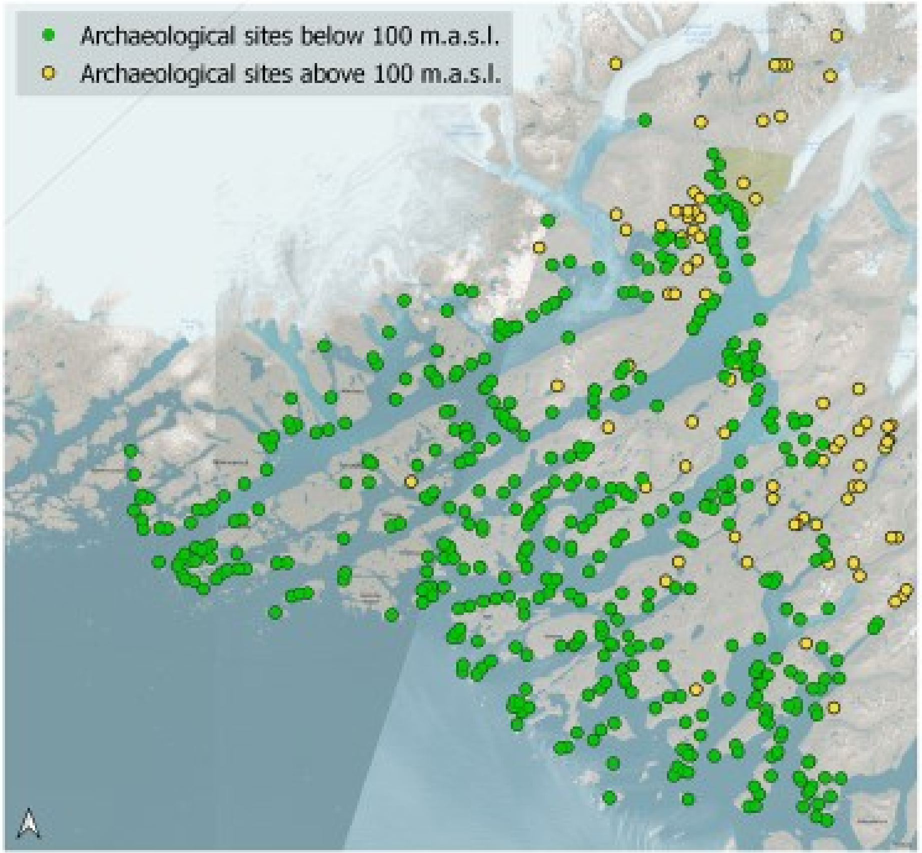

Supplementary Fig. S7: Total number of archaeological sites in the study area based on data from the Greenland national database of cultural heritage monuments and properties 'Nunniffit'.

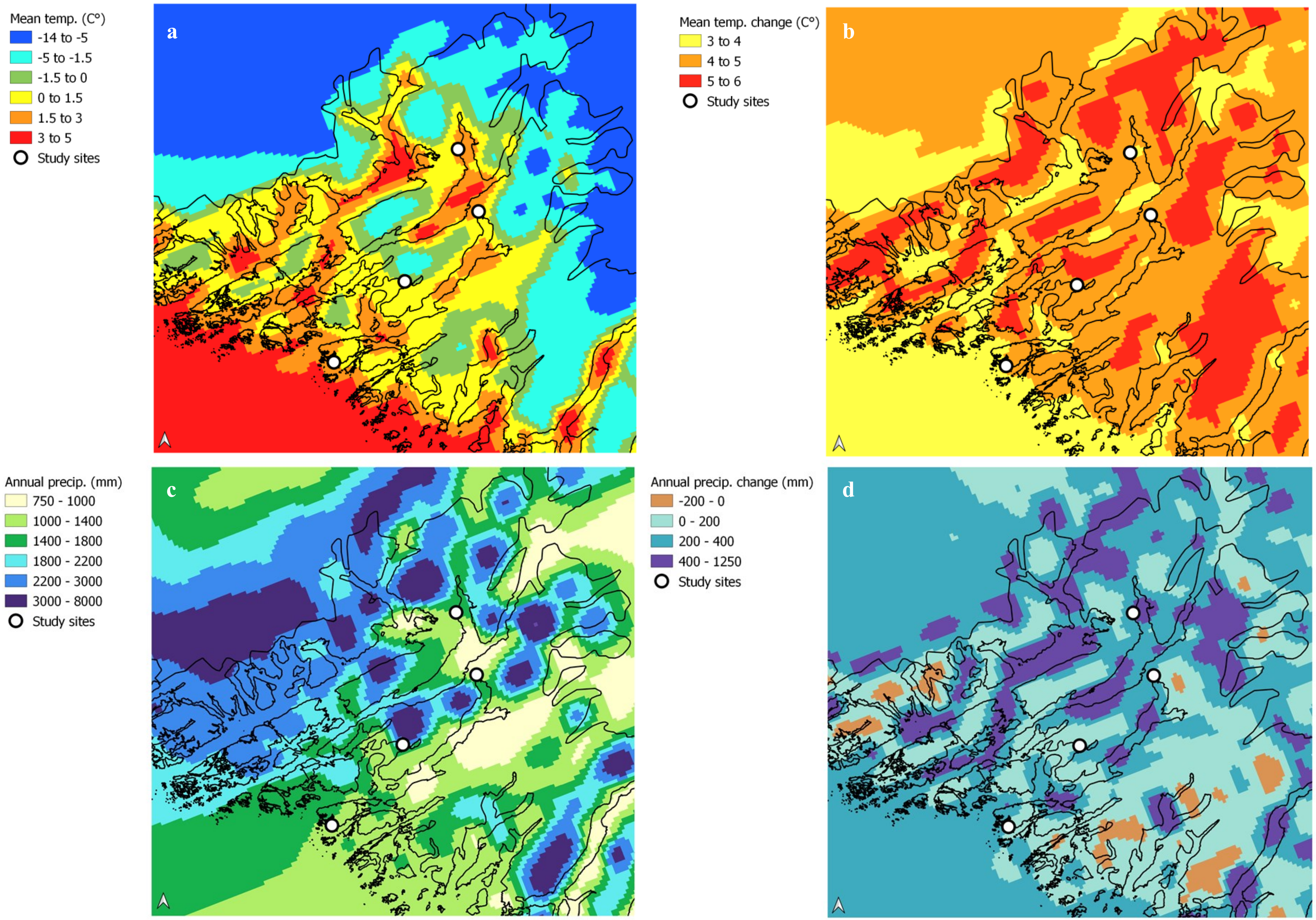

Supplementary Fig. S8: Modelled regional data from HIRHAM5 driven with the EC-Earth3 model. (**a**) Yearly mean air temperatures from 2001-2020. (**b**) Modelled change in yearly mean air temperatures from 2001-2020 to 2081-2100 based on the SSP5-8.5 scenario. (**c**) Yearly mean sum of precipitation from 2001-2020. (**d**) Modelled change in yearly sum of precipitation from 2001-2020 to 2081-2100 based on the SSP5-8.5 scenario.

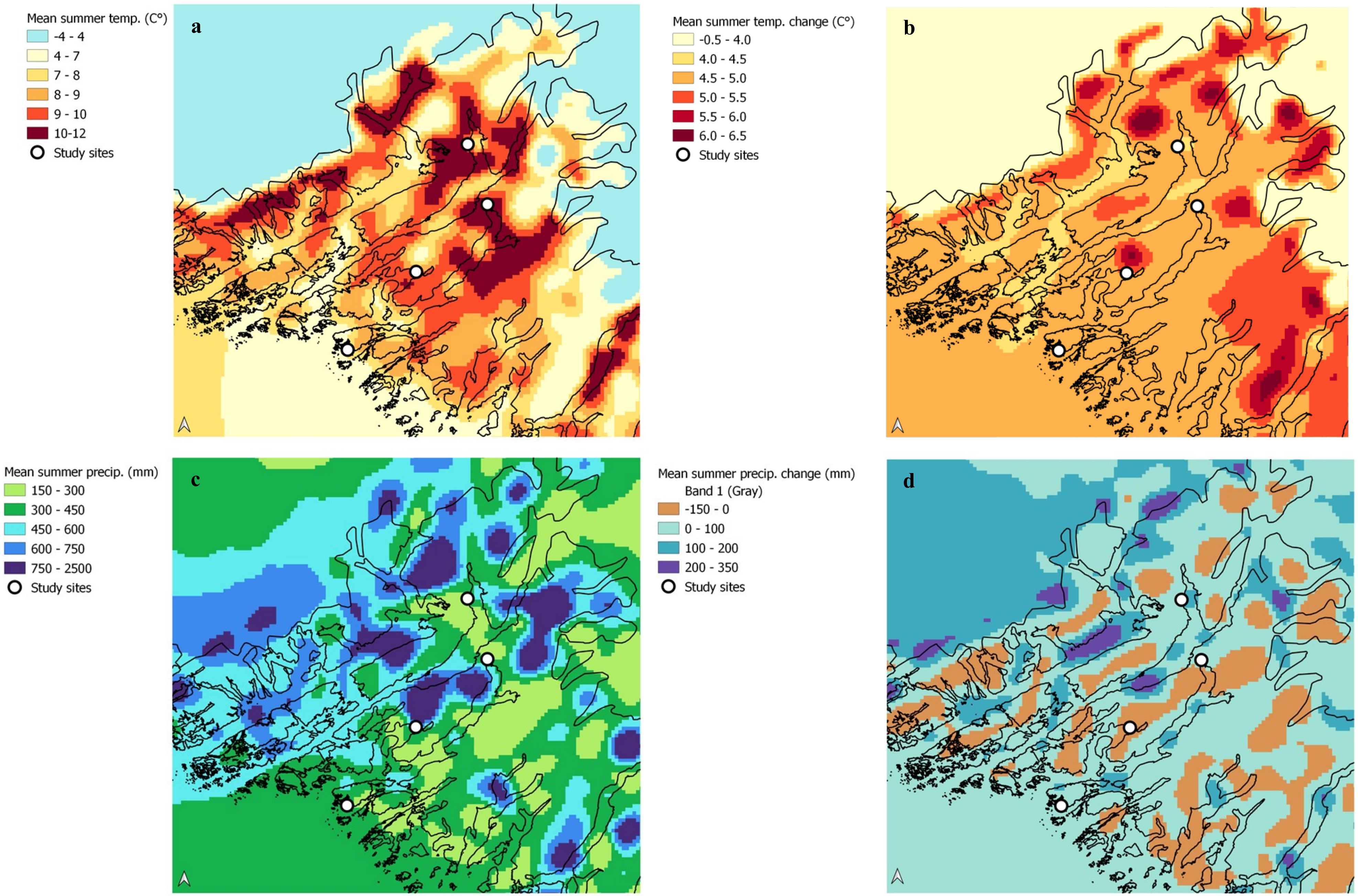

Supplementary Fig. S9: Modelled regional data from HIRHAM5 driven with the EC-Earth3 model. (**a**) Mean summer air temperatures from 2001-2020. (**b**) Modelled change in mean summer air temperatures from 2001-2020 to 2081-2100 based on the SSP5-8.5 scenario. (**c**) Mean sum of precipitation during summer from 2001-2020. (**d**) Modelled change in the sum of precipitation during the summer from 2001-2020 to 2081-2100 based on the SSP5-8.5 scenario.

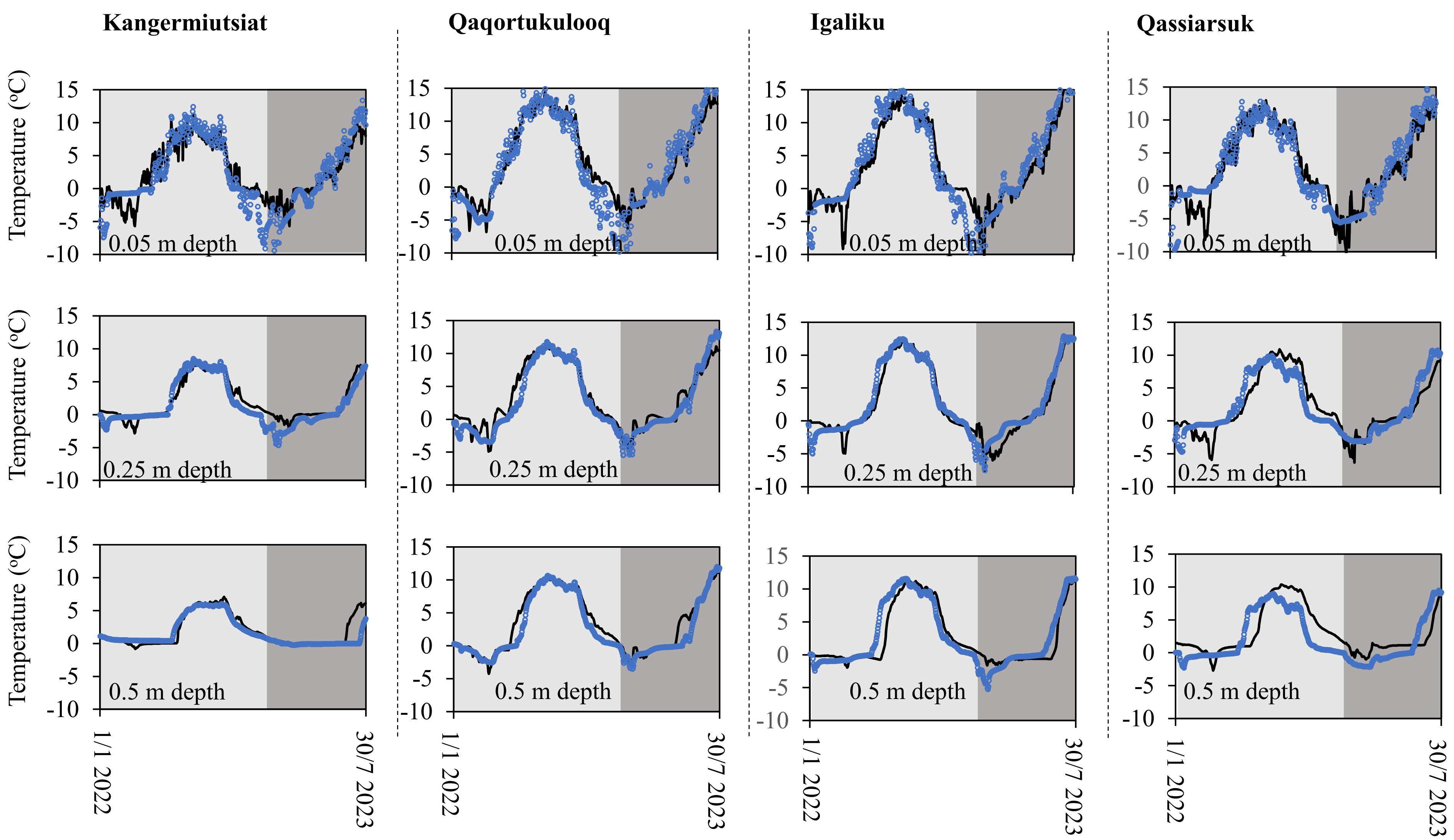

Supplementary Fig. S10 Simulated (blue) and measured (black) soil temperatures at the study sites from 1. January 2022 to 30. July 2023. The light grey areas indicate the calibration period and dark grey areas the validation period.

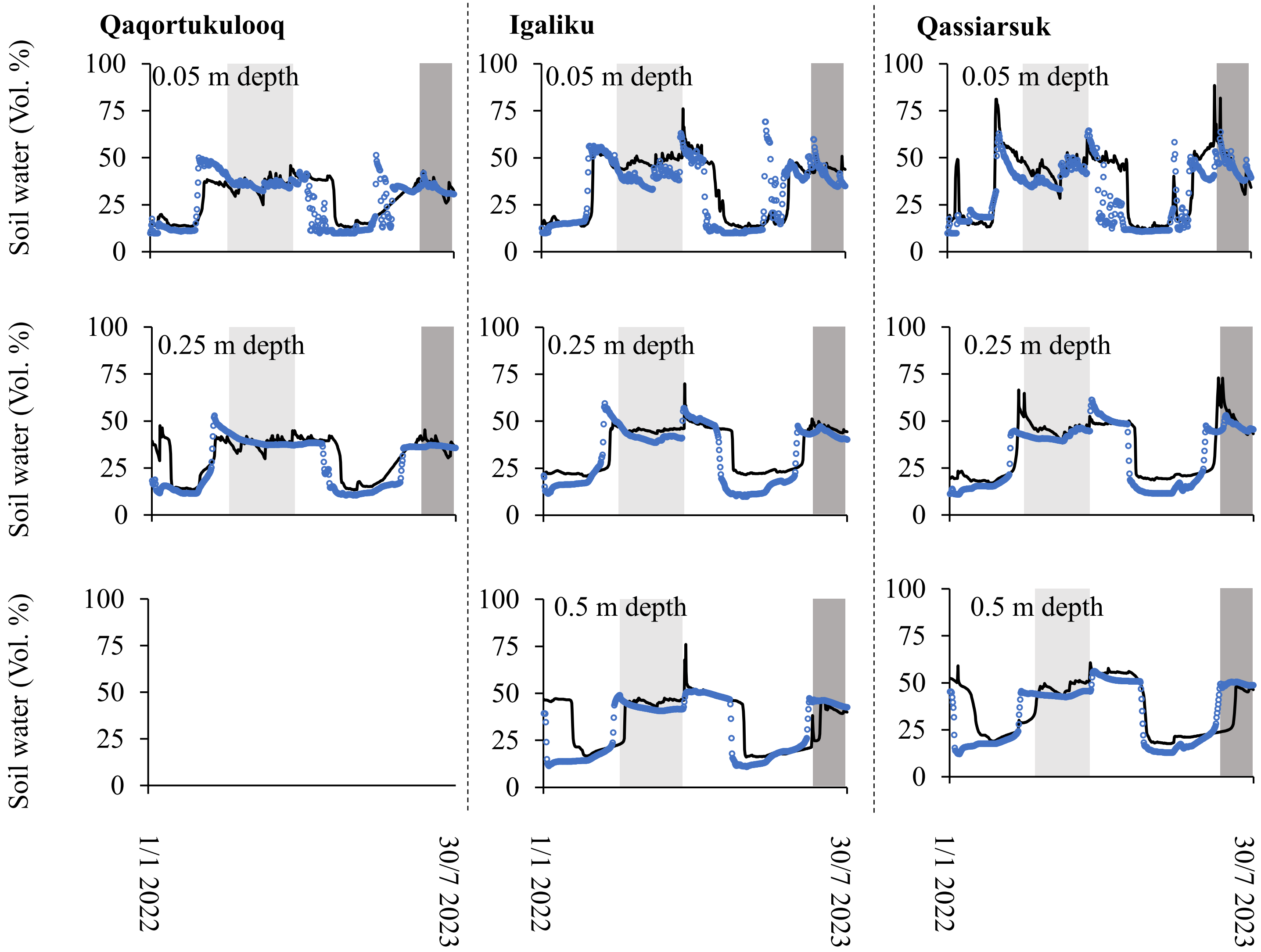

Supplementary Fig. S11: Simulated (blue) and measured (black) soil water contents from 1. January 2022 to 30. July 2023. The light grey areas indicate the calibration period and dark grey areas the validation period.

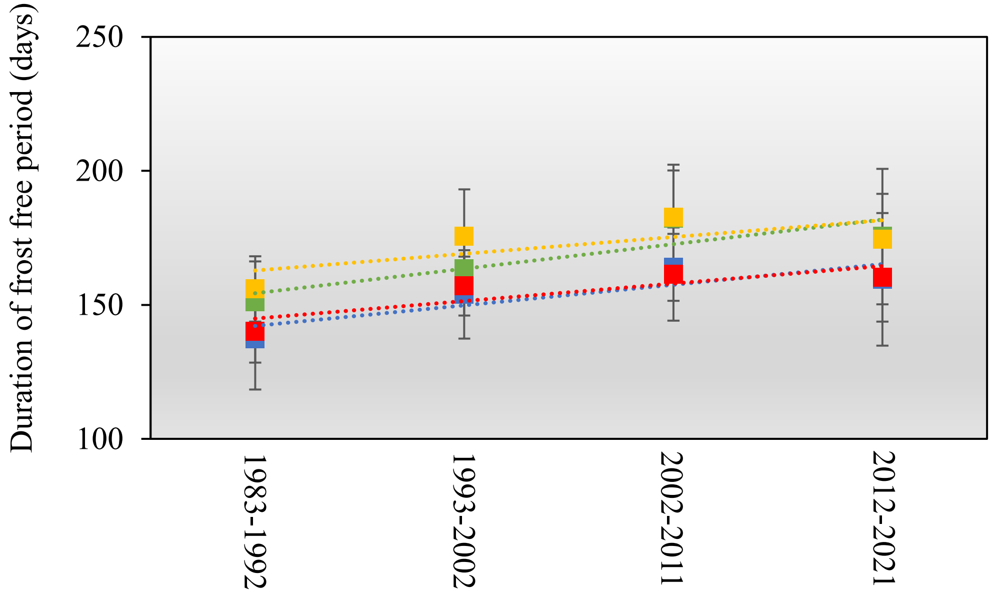

Supplementary Fig. S12 Modelled 10-year means of the duration of the frost-free period in 0.25 m depths at Kangermiutsiat (blue), Qaqortukulooq (green), Igaliku (yellow) and Qassiarsuk (red). Vertical bars show ± 1 s.d.

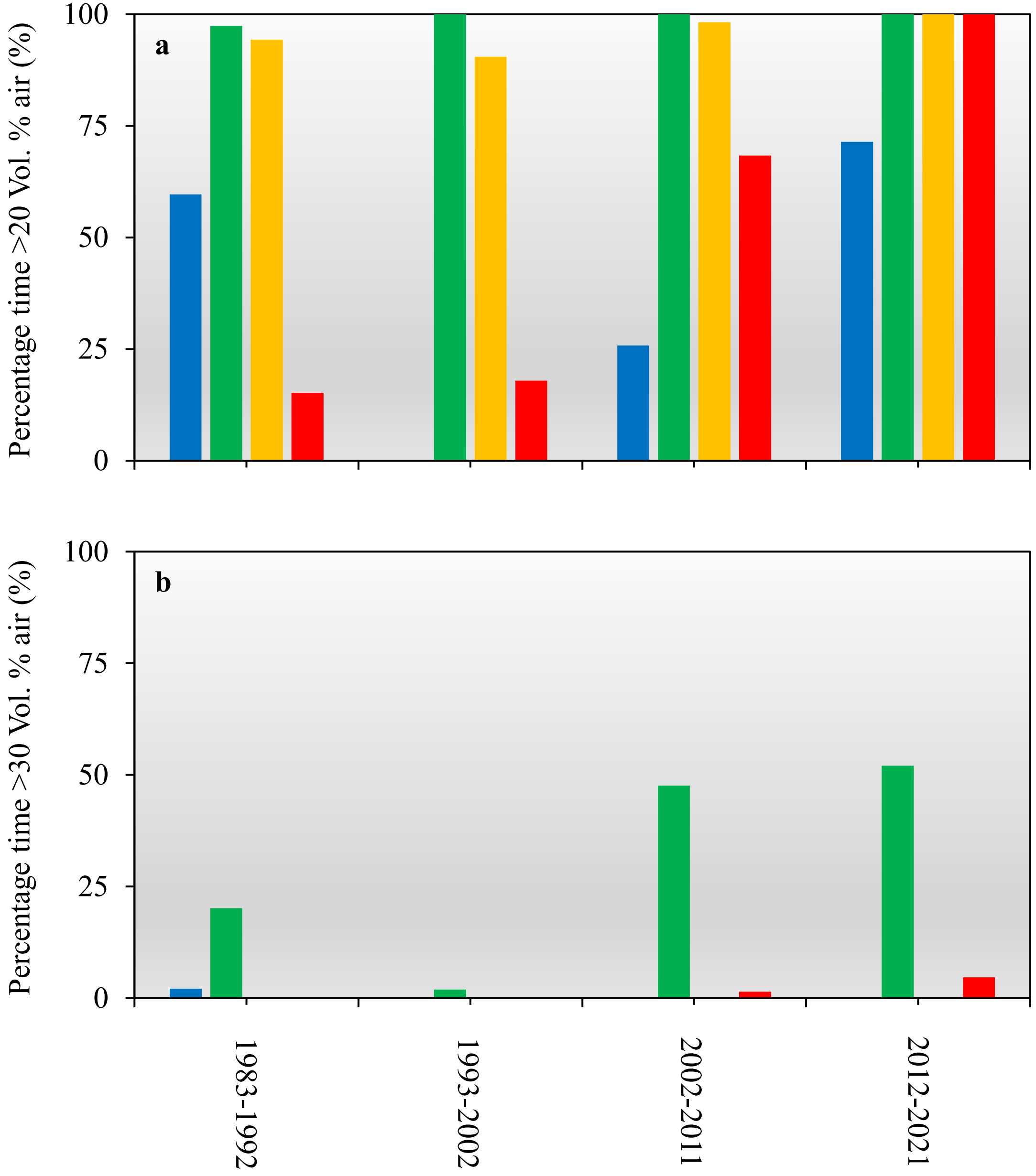

Supplementary Fig. S13: Modelled 10-year means of the duration of the percentage of time the soil water content is more than 20 Vol.% (**a**) and 30 Vol.% (**b**) below saturation during July and August (100% = 62 days). The data represents the conditions in 0.25 m at Kangermiutsiat (blue), Qaqortukulooq (green), Igaliku (yellow) and Qassiarsuk (red). Vertical bars show ± 1 s.d.

Site Name	NKAH #	Cultural phase	Test pit dimensions	Age estimate of cultural layers	Site description and soil character from test pit
**Kangermiutsiat**	2116	Historic/early modern Inuit	.5x.5 m	Late 19th – early 20th century, possibly older	An Inuit winter settlement, possibly in use up through the early 20th century. Dense vegetation covered the site. Lower soil horizons consisted of very dark brown/black greasy loam with sandy inclusions. Preservation of wood, bone and shell was extremely poor from ca. 15-40 cm below the surface.
**Qaqortukulooq (Ø83)**	4427	Norse	.5x.5 m	ca. 1000-1450 CE	Location of the Hvalsey church and farm. Last mention of the church in medieval sources mentions a wedding performed at the church in 1408. Test pit was placed several meters below the farmhouse ruins on the south facing slope. Poor preservation and very few artifacts recovered. Cultural layers were very shallow and diffuse comprising of compact and mottled sandy and silty loams. Sterile coarse sandy soil was encountered at ca. 40 cm below the surface, which may also explain the low soil moisture content resulting in poor preservation conditions at this location.
**Igaliku (Ø49)**	2252	Norse	1x2 m	ca. 1000-1450 CE	A small Norse farmstead located on a flood plain a few kilometers to the northeast of Igaliku. Because of the low elevation and periodic flooding, much of the upper cultural layers were absent when the area was excavated in 2021. Preservation was poor with a few metal and soapstone artifacts collected. A few small pieces of bone material in very poor condition were also recovered.
**Qassiarsuk (Ø29a)**	2230	Norse	.5x.5 m	ca. 1000-1450 CE	Large and important Norse farm complex (Brattahlíð, northern farm) with 21 registered features connected to the site. Test pit was dug in what was believed to be an unexcavated portion of the midden in front of the farmhouse, however we believe the area has experienced many disturbances from both farming activities and previous archaeological investigations over the past 100+ years. The test pit was placed on a graduated slope below the house leading down to the gravel road. The midden layers we encountered can be generally described as a friable dark brown silty loam with poor preservation, however archaeologists from the NKA working at the site in 2023 report isolated portions of the midden where wood preservation is still quite good (Michael Nielsen, personal communication 2023). This suggests preservation may be variable and influenced by local factors such as terrain and hydrology.

Supplementary Table S1: Study site informations.

Kangermiutsiat
Calibration period

Depth (m)	N	r^2	Regression Lines	Mean Error (y-x)
0.05	365	0.80	Y = 1.08x - 0.70	-0.47
0.25	365	0.88	Y = 1.07x - 0.37	-0.20
0.50	365	0.94	Y = 0.84x + 0.42	0.03

Kangermiutsiat
Validation period

Depth (m)	N	r^2	Regression Lines	Mean Error (y-x)
0.05	211	0.92	Y = 1.42x - 0.81	-0.15
0.25	211	0.88	Y = 0.99x - 0.99	-0.99
0.50	211	0.35	Y = 0.20x - 0.06	-0.92

Qaqortukulooq
Calibration period

Depth (m)	N	r^2	Regression Lines	Mean Error (y-x)
0.05	365	0.92	Y = 1.15x – 1.91	-1.27
0.25	365	0.95	Y = 1.05x - 1.13	-0.92
0.50	365	0.94	Y = 0.97x - 0.53	-0.65

Qaqortukulooq
Validation period

Depth (m)	N	r^2	Regression Lines	Mean Error (y-x)
0.05	211	0.94	Y = 1.26x – 0.40	0.46
0.25	211	0.96	Y = 1.16x - 0.55	-0.16
0.50	211	0.91	Y = 1.01x - 0.64	-0.63

Igaliku (Ø49)
Calibration period

Depth (m)	N	r^2	Regression Lines	Mean Error (y-x)
0.05	365	0.82	Y = 1.10x - 0.60	-0.80
0.25	365	0.93	Y = 1.02x + 0.07	0.12
0.50	365	0.81	Y = 0.95x + 0.22	0.04

Igaliku (Ø49)
Validation period

Depth (m)	N	r^2	Regression Lines	Mean Error (y-x)
0.05	211	0.94	Y = 1.13x + 0.38	-0.80
0.25	211	0.93	Y = 0.97x + 0.75	0.71
0.50	211	0.86	Y = 1.10x + 0.17	0.30

Qassiarsuk
Calibration period

Depth (m)	N	r^2	Regression Lines	Mean Error (y-x)
0.05	365	0.82	Y = 0.90x + 0.04	-0.17
0.25	365	0.78	Y = 0.80x + 0.34	-0.20
0.50	365	0.63	Y = 0.75x - 0.19	-1.17

Qassiarsuk
Validation period

Depth (m)	N	r^2	Regression Lines	Mean Error (y-x)
0.05	211	0.94	Y = 1.10x + 0.71	0.83
0.25	211	0.91	Y = 1.20x + 0.43	0.64
0.50	211	0.74	Y = 1.05x - 1.09	-0.36

Supplementary Table S2: Statistics on the agreement between observed (x) and simulated soil temperatures (y) for the calibration period from 1. January 2022 to 31. December 2022 (left) and for the test period from 1. January 2023 to 30. July 2023.

Qaqortukulooq
Calibration period

Depth (m)	N	r^2	Regression Lines	Mean Error (y-x)
0.05	122	0.72	Y = 0.34x + 23.18	-0.74
0.25	122	0.14	Y = -0.18x + 45.46	-0.58

Qaqortukulooq
Validation period

Depth (m)	N	r^2	Regression Lines	Mean Error (y-x)
0.05	60	0.57	Y = 0.54x + 15.42	-0.32
0.25	60	0.12	Y = 0.05x + 34.83	-0.56

Igaliku (Ø49)
Calibration period

Depth (m)	N	r^2	Regression Lines	Mean Error (y-x)
0.05	122	0.35	Y = 0.83x + 0.31	-7.91
0.25	122	0.47	Y = 0.97x - 2.02	-3.30
0.50	117	0.64	Y = 0.41x + 22.91	-4.31

Igaliku (Ø49)
Validation period

Depth (m)	N	r^2	Regression Lines	Mean Error (y-x)
0.05	60	0.07	Y = 0.76x + 6.15	-4.46
0.25	60	0.90	Y = 1.38x - 20.4	-3.21
0.50	50	0.69	Y = 0.43x + 26.27	3.07

Qassiarsuk
Calibration period

Depth (m)	N	r^2	Regression Lines	Mean Error (y-x)
0.05	122	0.65	Y = 1.11x - 7.92	-2.91
0.25	122	0.42	Y = 1.14x - 8.60	-2.39
0.50	108	0.47	Y = 0.47x + 21.48	-4.10

Qassiarsuk
Validation period

Depth (m)	N	r^2	Regression Lines	Mean Error (y-x)
0.05	60	0.89	Y = 0.61x + 18.20	0.97
0.25	60	0.29	Y = 0.19x + 37.99	-2.87
0.50	45	0.48	Y = 0.54x + 23.97	2.86

Supplementary Table S3: Statistics on the agreement between observed (x) and simulated soil water contents (y) for the frost-free period in 2022 (calibration period) and the frost-free period in 2023 until 30. July (validation period).

Data type	Kangermiutsiat	Qaqortukulooq	Igaliku	Qassiarsuk
**Air temperature**	S-THB-M002 Temp/RH Sensor (HOBO, Bourne, Massachusetts, US)	S-THB-M002 Temp/RH Sensor (HOBO, Bourne, Massachusetts, US)	S-THB-M002 Temp/RH Sensor (HOBO, Bourne, Massachusetts, US)	S-THB-M002 Temp/RH Sensor (HOBO, Bourne, Massachusetts, US)
**Rain**	*Data from an official meteorological station located in Qaqortoq 10 km to the East was used	S-RGB-M002 Tipping Bucket Raingauge (HOBO, Bourne, Massachusetts, US)	S-RGB-M002 Tipping Bucket Raingauge (HOBO, Bourne, Massachusetts, US)	S-RGB-M002 Tipping Bucket Raingauge (HOBO, Bourne, Massachusetts, US)
**Soil temperature**	S-TMB-M002 temperature probes (HOBO, Bourne, Massachusetts, US)	S-TMB-M002 temperature probes (HOBO, Bourne, Massachusetts, US)	S-TMB-M002 temperature probes (HOBO, Bourne, Massachusetts, US)	S-TMB-M002 temperature probes (HOBO, Bourne, Massachusetts, US)
**Soil moisture**	Theta Probe (Delta-T Devices Ltd, Cambridge, UK)	Theta Probe (Delta-T Devices Ltd, Cambridge, UK)	Theta Probe (Delta-T Devices Ltd, Cambridge, UK)	Theta Probe (Delta-T Devices Ltd, Cambridge, UK)

Table S4: Overview of the environmental parameters monitored at the four study sites and the equipment used.
